# Fast interlayer exciton drift driven by lattice reconstruction in a van der Waals heterobilayer

**DOI:** 10.1038/s41563-026-02688-2

**Published:** 2026-07-29

**Authors:** Fedele Tagarelli, Edoardo Lopriore, Cristian de Giorgio, Daniel Erkensten, Raül Perea-Causín, Samuel Brem, Kenji Watanabe, Takashi Taniguchi, Ermin Malic, Andras Kis

**Affiliations:** 1 Institute of Electrical and Microengineering, École Polytechnique Fédérale de Lausanne (EPFL), Lausanne, Switzerland; 2 Institute of Materials, École Polytechnique Fédérale de Lausanne (EPFL), Lausanne, Switzerland; 3 Department of Physics, Philipps-Universität Marburg, Marburg, Germany; 4 Department of Physics, Stockholm University, Stockholm, Sweden; 5 Research Center for Functional Materials, National Institute for Materials Science, Tsukuba, Japan; 6 International Center for Materials Nanoarchitectonics, National Institute for Materials Science, Tsukuba, Japan

**Keywords:** Optical materials and structures, Nanophotonics and plasmonics, Two-dimensional materials

## Abstract

Modern short-scale information transmission mainly relies on dissipative charge transport, with electrons scattered by defects and phonons, leading to significant power losses. By contrast, excitons—charge-neutral quasiparticles—offer a playground for electro-optical energy-efficient information transduction and processing owing to their extended lifetimes, charge neutrality and efficient electrostatic control. In this work, we report the observation of fast exciton transport in a van der Waals heterostructure over distances exceeding 10 µm, constrained only by the heterostructure finite size. We observe the presence of excitonic potential ramps that leads to long-range rapid exciton drift and enables rapid dilution of the initial exciton population. Our measurements reveal fast exciton propagation, with interlayer exciton drift velocities of approximately 2.66 × 10^4^ m s^−1^, within a transport regime that remains robust across a wide range of exciton densities and temperatures up to 150 K. Our work opens avenues for the development of high-speed, energy-efficient excitonic devices, such as field-effect switches and modulators.

## Abstract (web summary)

Time-resolved measurements of interlayer exciton transport in van der Waals heterobilayers show that lattice reconstruction generates potential energy gradients, driving rapid exciton drift towards low-energy recombination sites.

## Main

The shift of long-range information transmission from the electrical to the optical domain has been a highly effective technological booster in the past, enabling the fast exchange of information with a global reach. Information is still exchanged through electrical charges over shorter length scales, that is, within single or multiple integrated circuits. This accounts for the majority of dissipated power in most technological applications, from data centres to mobile computing^1^. Therefore, significant effort has recently been focused on achieving efficient heterogeneous integration of photonic and electronic material platforms to further reduce dissipated power and increase chip-level computing bandwidth^2,3^.

Integrated excitonic interconnects, which transport excitons, offer the potential to reduce dissipated thermal power thanks to their charge-neutral nature and can scale down more than photonic interconnects^4^. Indirect or interlayer excitons (IXs), hosted in two-dimensional (2D) material heterostructures, are particularly suitable for this purpose, since the spatial separation of charges in different material layers allows efficient electrostatic control via the quantum confined Stark effect^5–7^. IXs also feature long lifetimes, making them promising candidates for information carriers^8,9^, while the advent of 2D materials has enabled breakthroughs, such as the demonstration of a room-temperature excitonic transistor^10^. All these features make the study of long-propagating and electrostatically controllable quasiparticles a promising avenue for the development of excitonic interconnects.

Previous studies of superfluid intralayer exciton transport have shown that excitons can travel at high speeds, up to 6% of the speed of light, and over distances of 60 µm (ref. ^11^). However, theoretical analysis indicates that superfluidity alone does not explain these speeds, which may be influenced by light waveguiding effects^12^. Moreover, only a small fraction of excitons reach these high speeds, with the majority recombining near the excitation region. However, extended spatial diffusion (more than 10 µm) of indirect excitons in steady-state conditions has also been observed^5,13–15^, showing highly inhomogeneous spatial patterns caused by rotational and lattice disorder in a heterogeneous moiré superlattice^16^. Nevertheless, exciton transport in van der Waals heterostructures has been studied through sub-nanosecond time-resolved measurements only at short distances, typically less than 5 µm from the laser excitation region^6,17–20^.

In this work, we investigate the time-resolved transport properties of IXs propagating in highly inhomogeneous spatial features in an ultra-smooth van der Waals heterostructure. Following optical excitation of IXs in our rhombohedral (R)-type heterobilayer stack, we observe the emergence of remote bright radiative recombination sites across the entire device. We analyse the spectroscopic features of the emitted photoluminescence (PL) at the recombination spots, as well as the reflected signal across the whole device. Our measurements indicate that excitons initially generated at the excitation spot are transported via lattice reconstruction-induced potential ramps to the low-energy recombination sites, in which they are trapped and recombine. Furthermore, time-resolved PL (TRPL) measurements allow us to identify the arrival rate of excitons at the remote emission sites, enabling us to estimate an average drift velocity for interlayer excitons in these potential ramps. Specifically, we estimate IX drift velocities and field-effect mobilities of *v*_d_ ≈ 2.66 × 10^4^ m s^−1^ and *μ*_f_ ≈ 1,320 ± 70 cm^2^ V^−1^ s^−1^, respectively. Furthermore, we use finite-element modelling simulations with the estimated transport properties and find qualitative agreement with our observations. Varying the optical excitation power results in a drastic change in the emitted PL of the recombination sites, confirming the influence of dipolar repulsive interactions and exciton–exciton annihilation on the density-dependent nature of IX transport^5,6,17,21^. Finally, we show that temperature serves as an effective control for the observed transport mechanism. Phonons disrupt long-range propagation above 150 K, in which transport becomes dominated by thermally activated hopping^18,22^.

## Device structure and IX energy landscape

**Fig. 1:**
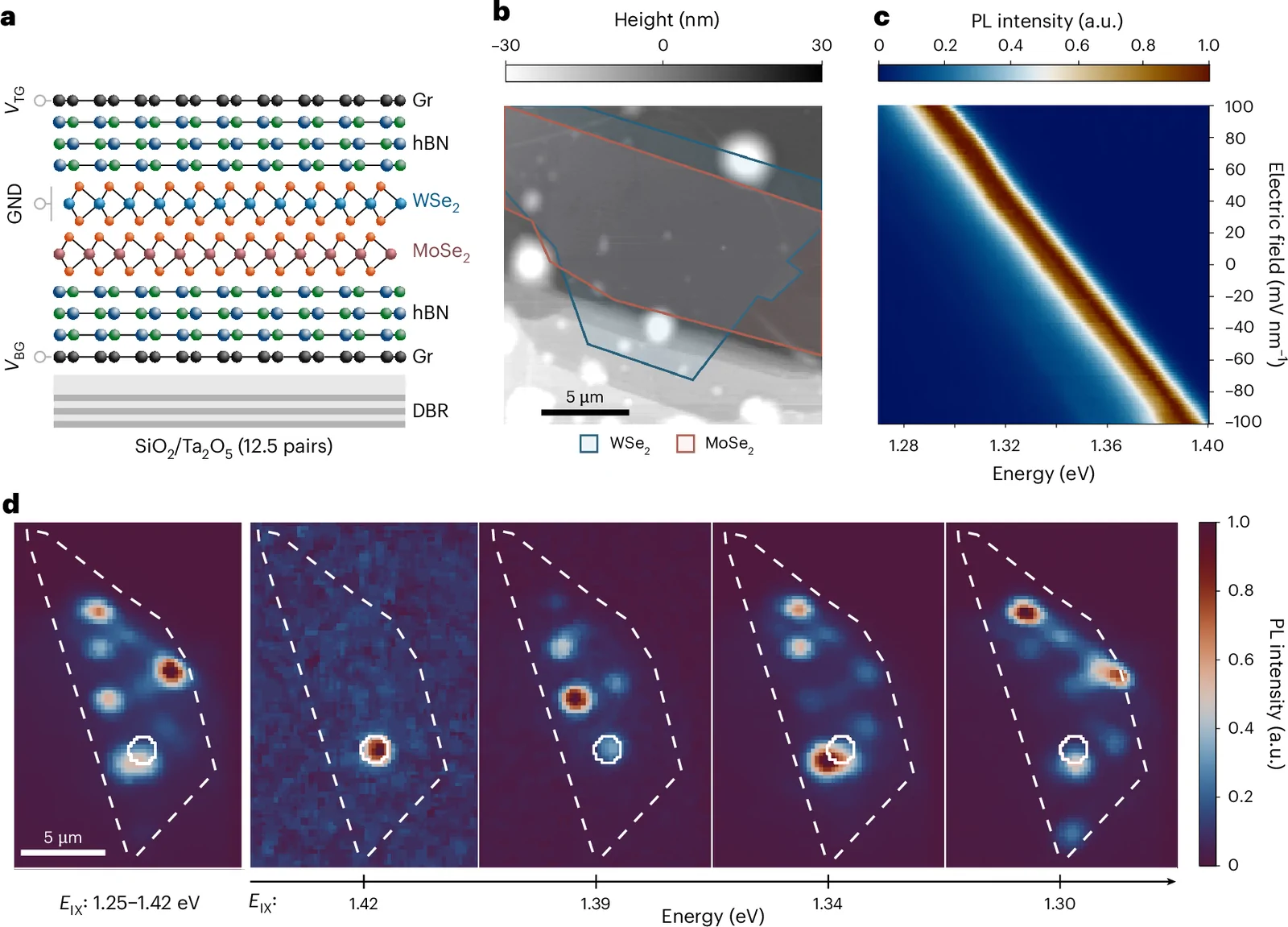
Device schematics and IX energy landscape. **a**, Schematic structure of the device. **b**, Atomic force microscopy image of device A. The overlays show the partial contours of the TMDCs within the heterostructure. **c**, Representative Stark effect map of interlayer exciton as a function of the vertical electric field. **d**, Hyperspectral galvo-scanning maps acquired by keeping the laser excitation spot in a fixed position (white solid circle), while the detection area was scanned over sample surface by means of galvo-scanning mirrors. The maps correspond to integrated intensities within the specified energy range for leftmost map and a ±1 meV range centred on the values noted on the horizontal axis for the remaining maps. The white dashed overlay shows the heterostructure region. *V*_BG_, bottom gate voltage; *V*_TG_, top gate voltage; GND, electrical ground; DBR, distributed Bragg reflector; au, arbitrary units.

We realize long-range interlayer exciton transport in a fully hexagonal boron nitride (hBN)-encapsulated MoSe_2_/WSe_2_ heterobilayer on a distributed Bragg reflector substrate. The heterobilayer features R-type stacking with a twist angle of *Θ*_twist_ ≈ 3.4 ± 0.7°, as confirmed by second-harmonic generation measurements (Supplementary Note 1). A schematic view of the structure under study is shown in Fig. 1a. In particular, our device is characterized by a very smooth morphology, as confirmed using atomic force microscopy (Fig. 1b). The absence of interfacial bubbles allows us to rule out any alteration of the excitonic potential environment and, therefore, any effect of out-of-plane strain on IX transport^23^. We confirm the interlayer nature of the excitonic species by spectroscopically resolved PL measurements and as Stark shift measurements shown in Fig. 1c from which we estimate a static vertical dipole moment of ~0.5 nm (Supplementary Note 2), which is in agreement with previous reports^21,24–26^. We locally excite interlayer excitons and collect the emitted PL from any point of the heterostructure using the galvo-scanning technique (Supplementary Note 11). In Fig. 1d, we show the PL emitted by the sample, for different regions of the emission spectrum, showing a highly inhomogeneous emission pattern. In particular, the spatial pattern features several bright recombination sites that are not continuously linked to the laser excitation area by bright PL emission. However, the analysis of the energy-dependent PL intensity shows that only higher-energy PL is emitted from the excitation spot (Fig. 1d), while other recombination sites emit PL at lower energies (Fig. 1d). The full dataset is provided in Supplementary Movie 1. We observe good agreement with previous reports^16^ in which a large lattice reconstruction period has been measured in WSe_2_/MoSe_2_ heterobilayers at the edges of heterostructures, spatially correlated with brighter recombination sites in R-stacked heterobilayers. Despite negligible morphological nonidealities in this device, we detect significant energy and intensity variations in the emitted PL between different bright recombination sites, which can be attributed to their distinct local lattice reconstruction periodicity^16^.

## Fast interlayer exciton transport

Space-resolved and time-resolved imaging of IXs serves a fundamental role in understanding the forces driving the movement of these quasiparticles. As shown in Supplementary Note 3, we observe the aforementioned remote bright recombination sites within a wide range of excitation densities with comparable behaviour, either below or above the Mott transition^17,27^. Furthermore, by modelling our excitonic platform with finite element analysis (FEA), we are able to show that fast exciton transport dilutes the generated exciton density to levels below the Mott transition in a few hundreds of picoseconds (Supplementary Note 9); therefore, we hereafter focus on the high-density regime for the sake of improved signal-to-noise ratio.

**Fig. 2:**
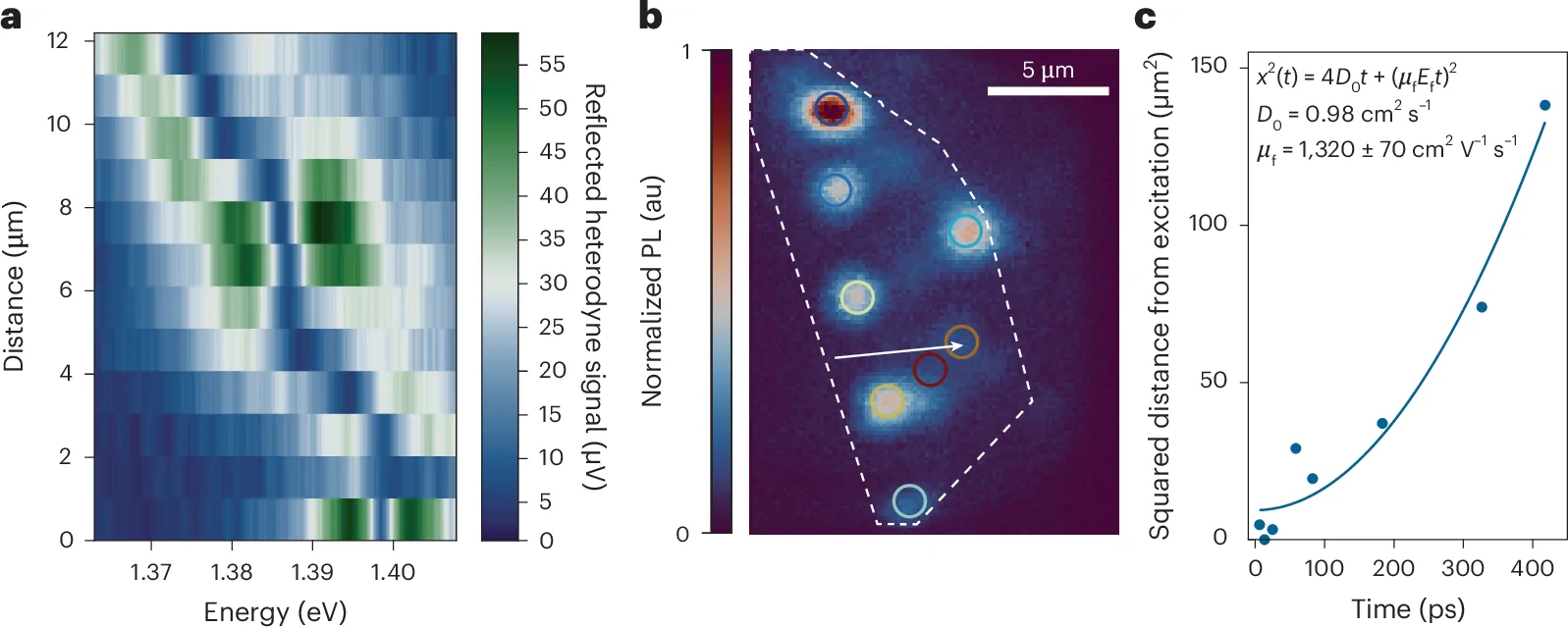
Time-resolved interlayer exciton propagation. **a**, Spatially resolved electro-modulation spectroscopy signal, obtained along the arrow in panel **b**, showing the energy derivative of the reflectivity peak of IXs. Supplementary Fig. 13 further shows a 2D spatial map of the main IX energy absorption dip obtained throughout a wide region of the heterostructure area. **b**, Spatially resolved IX PL imaging using galvo-scanning. The coloured circles represent the collection regions for colour-matched TRPL signals representing the distance from the laser excitation spot. The white arrow indicates the path of the electro-modulation spectroscopy signal shown in part **a**. For reference, the same arrow is located in the electro-modulation spectroscopy map in Supplementary Fig. 13. **c**, Squared distance of the collection spot from the laser excitation spot as a function of the average rise time. We estimate the rise time of the TRPL signals for each position by the probabilistic fitting approach described in Supplementary Note 5. The data is fitted following the equation reported as inset.

To explain such large exciton speeds, we first investigate the ground-state excitonic energy landscape across the heterostructure via electro-modulation spectroscopy, as previously shown by Barré et al.^26^. In Fig. 2a, we show a linear spatial scan, where we clearly resolve a shift of the exciton energy of roughly 25 meV across 12 µm. Notably, this is comparable in magnitude with previously reported electrostatically induced potential ramps^28^. Furthermore, in Supplementary Note 8, we show the 2D spatial mapping and histogram of the interlayer exciton resonance energy, obtained by electro-modulation spectroscopy across the entire device region of interest, revealing an energy distribution that spans 40 meV within the double-gated heterostructure.

To characterize the spatial and temporal behaviour of fast IX transport, we use a galvo-scanning time-correlated single-photon counting PL technique (Supplementary Note 11). By exciting the same spot as shown in Fig. 1d, we are creating a high-potential excitonic density that can then drift towards neighbouring lower-potential regions. Figure 2b presents the time-integrated PL spatial map, where each recombination site is colour-coded with different circular overlays. These indicate the locations from which we collect the time traces with relative distance from the excitation region as shown in Supplementary Fig. 9. The TRPL signals from the various recombination sites show different rise trends based on their respective locations. To estimate the rise time of each TRPL signal, we fit the experimental data with an arbitrary number of decay and rise rates convoluted with the instrument response function and optimized by a basis pursuit denoising algorithm^29^ (for further details, see Supplementary Note 5). We show in Fig. 2c the amplitude-weighted average of the rise times for each recombination site plotted as a function of their respective distance from the excitation spot. While measurements taken near the excitation spot show rise times comparable with the system resolution limit of 50 ps (see Supplementary Note 11), the TRPL signals from more distant regions show a clear increasing trend. We estimate the drift velocity and field-effect mobility of IX propagation using a drift-diffusion model with ${x}^{2}\left(t\right)={x}_{0}^{2}+{4D}_{0}t+{({\mu }_{{\rm{f}}}{E}_{{\rm{f}}}t)}^{2}$, where *x*(*t*) is the propagation distance, *x*_0_ is the initial position, *D*_0_ is the diffusion coefficient, *μ*_f_ is the exciton field-effect mobility, and *E*_f_ is the effective in-plane field driving the IXs. The latter is estimated by linearly interpolating the local potential variations induced by lattice reconstruction, as obtained from electro-modulation spectroscopy measurements (Fig. 2a and Supplementary Note 9). On the basis of the line scan in Fig. 2a and of the map of Supplementary Fig. 13, for our estimation, we extract an average potential spread of ~25 meV, thus giving an average *E*_f_ value. Regarding the intrinsic diffusivity, we use established results on IX transport in MoSe_2_/WSe_2_ (ref. ^17^) with *D*_0_ ≈ 0.98 cm^2^ s^−1^, as it is not directly resolvable in our drift-dominated measurements. Fitting the aforementioned model, we find high drift velocities of *v*_d_ = *μ*_f_ *E*_f_ ≈ 2.66 × 10^4^ m s^−1^, corresponding to a field-effect mobility of *μ*_f_ ≈ 1,320 cm^2^ V^−1^ s^−1^. We note that such an approach assumes that the bright recombination sites are reached in an approximately isotropic way from the region of excitation. This is justified by the fact that our excitation region corresponds to a high-potential spot located in a central region of the heterostructure area (Fig. 1d), thus resulting in the outwards drift of IXs towards lower-energy regions throughout the sample.

To confirm the validity of our findings, we use FEA to solve the exciton continuity equation, obtaining results in good qualitative agreement with experimental data (Supplementary Note 9). Previous studies on similar systems have reported fast IX transport driven by exciton–exciton annihilation and repulsion^6,17,18^, or related to electron–hole plasma^17,27^, respectively. As mentioned at the beginning of this section, we exclude electron-hole plasma as an explanation for our observations even in the high-density regime. In fact, as detailed in Supplementary Note 9, our FEA shows that, even for high-density excitations, the Mott regime is maintained only for a few hundreds of picoseconds after the laser pulse.

We have performed energy-resolved and time-resolved PL studies at the excitation region via Streak camera measurements (see Methods and Supplementary Note 8), revealing the presence of both high-energy and low-energy exciton species with short (sub-nanosecond) and long (nanosecond) lifetimes, respectively. This indicates that high-energy IXs can escape the excitation region and navigate the potential landscape, thus appearing as short-lived species within the excitation region. However, lower-energy IXs do not travel across the heterostructure, and these IXs radiate mostly in the excitation region, being the main contributions to the collected confocal PL signal at this spot.

## The role of IX interactions on fast IX drift propagation

Type II interlayer excitons in van der Waals heterostructures are characterized by large static dipoles, as their constituent electrons and holes are spatially confined in different layers^21,24,30^. Therefore, interlayer exciton transport strongly depends on the initial exciton density^31,32^. In the low-density regime, diffusivity increases with the misalignment between the two constituting layers, attributed to shallower moiré traps^18,19^. At elevated exciton densities, dipolar repulsive interactions and exciton–exciton annihilation dominate IX transport^5,6,21^.

**Fig. 3:**
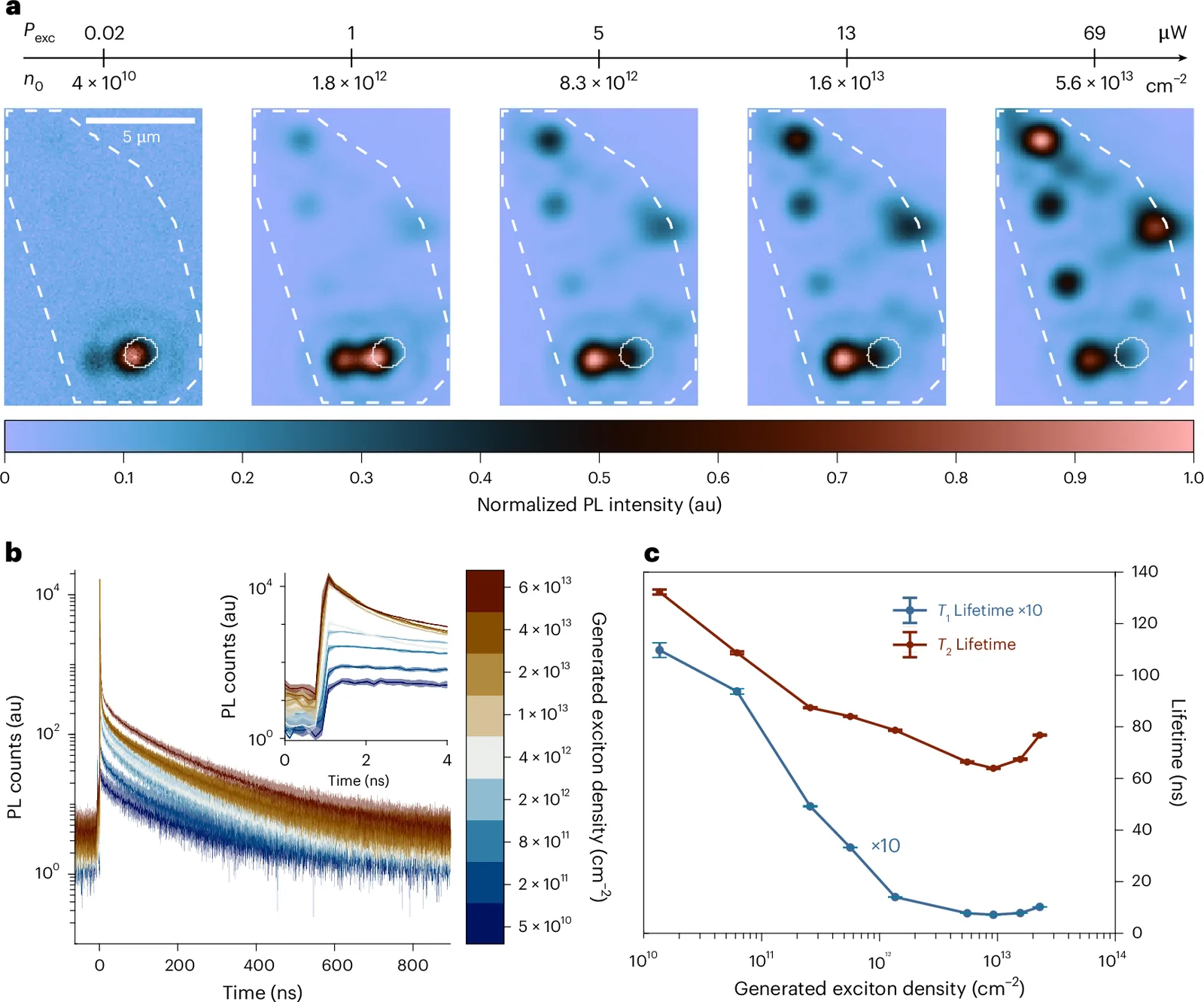
Density-dependent IX transport and dynamics. **a**, Density-dependent time-integrated PL imaging. The white contour outlines the heterostructure region within the field of view. The white circle indicates the position of the laser excitation. While at low-density regimes long-range IX transport seems to disappear, we find qualitative agreement with continuous wave power-dependent IX imaging from moderate to high generated exciton densities. In this case, the absence of long-range IX transport in the low-density regime can be attributed to various experimental trade-offs between the emitted PL and the system’s noise floor, which can hide the weak remotely emitted PL beneath the noise floor. **b**, TRPL emitted from the laser spot at different generated exciton densities. Inset: Initial time interval relative to the arrival of optical excitation. **c**, Estimated lifetime as a function of the generated exciton density, obtained by fitting a double exponential decay to the measured TRPL signal. The faster decay time is multiplied by ten. Error bars correspond to the standard deviation around the mean. Error bars correspond to the 95% confidence interval.

In the previous sections, we show time-resolved diffusion at high optical excitation regimes. Next, we investigate density-dependent IX transport by steady-state PL imaging. We observe long-range IX transport even at low optical pump power (Supplementary Movie 2), that is, *P*_exc_ <1 µW (Supplementary Note 3), corresponding to exciton densities *n*_IX_ < 10^12^ cm^−2^ (Supplementary Note 7). Furthermore, as the optical excitation power increases (Fig. 3a), the IX population increasingly occupies lower-energy remote recombination sites. This suggests that exciton–exciton annihilation and repulsive interactions further facilitate IX propagation but lead to slower net transport (Supplementary Note 10). At elevated excitation powers, PL intensities emitted from remote recombination sites surpass those from the local excitation spot (Fig. 3b), highlighting the high efficiency of this transport mechanism for radiatively recombining interlayer excitons. This effect is very stable and independent from the placement of the excitation spot within the heterostructure, as shown in Supplementary Note 3.

In Fig. 3c, we present the power-dependent IX lifetimes, estimated using a double exponential decay model, which reveal decreasing exciton lifetimes (both *T*_1_ and *T*_2_) as the optical excitation power is increased. This behaviour is often attributed to density-dependent second-order non-radiative recombination dynamics, such as exciton–exciton annihilation^33^. However, we observe that the density-dependent IX lifetime initially decreases and then plateaus for higher densities, which does not align with second-order non-radiative scattering mechanisms. Instead, the decrease of estimated lifetime can be explained by the rapid escape of IXs from the collection spot, which is usually counterbalanced by collecting PL emission from a large sample area, especially in studies where fast exciton movement cannot be neglected^34^. This rapid escape of exciton density upon excitation is further supported by the presence of higher PL signal from far-emitting spots in the time-integrated condition, while short timescales in TRPL measurements indicate higher peak intensities for spots closer to the excitation spot. While Fig. 3c indicates a faster escape speed from the excitation region with increasing density, galvo-scanning density-dependent time-resolved PL (Supplementary Note 10) reveals a slower average drift speed from the excitation region to the bright recombination sites for increasing generated exciton densities. This can be explained by increased phonon scattering because of heating effects with increasing fluence. In addition, with increasing exciton densities, we expect larger portions of the total generated population to move via other transport mechanisms characterized by smaller effective mobilities, such as exciton–exciton annihilation and repulsion^17^. We note that stronger optical excitations may also induce phonon wind or drag effects^35^, leading to halo-like excitonic distributions and faster transport with respect to conventional diffusion. However, these processes are limited to the speed of sound, which is orders of magnitude slower than the observed IX drift velocity.

## Temperature-dependent IX transport

**Fig. 4:**
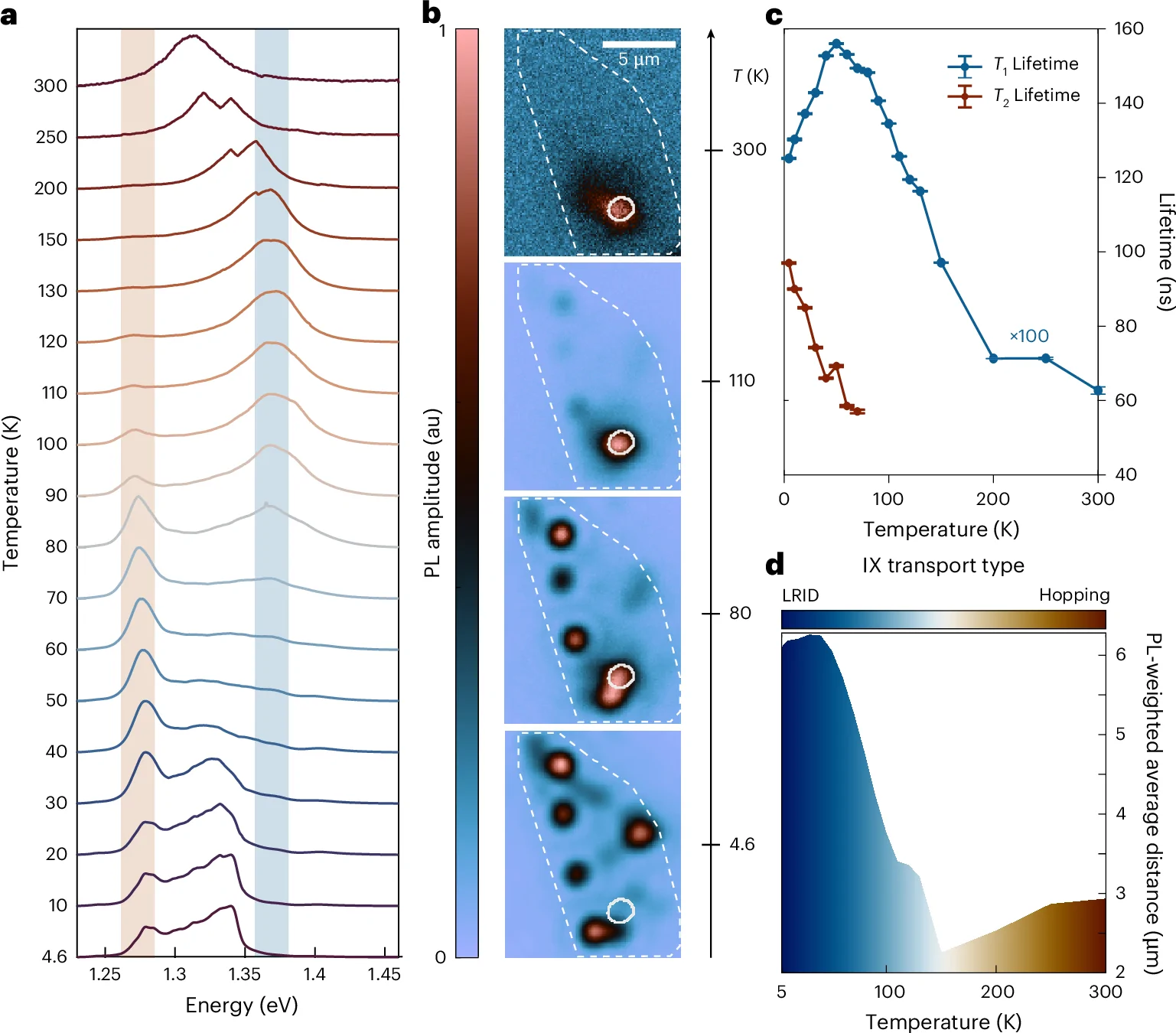
Temperature-dependent fast IX drift transport. **a**, Spectrally resolved PL emission normalized and offset from the laser spot as a function of system temperature. A clear exchange between the maximum intensity peaks is observed at 80 K. In agreement with previous reports, we ascribe the low-energy peak to localized moiré excitons, and the high-energy one to free excitons^22^. **b**, Time-integrated IX PL recorded at 300 K, 110 K, 80 K and 4.6 K from top to bottom, respectively. A rapid suppression of long-range IX transport is observed for temperatures above 110 K. **c**, Lifetime estimated from fitting a double exponential decay model to the temperature-dependent TRPL signal. The increase of the fast decay time for temperatures lower than 50 K indicates that *T*_1_ represents the fast depopulation of excitons from the laser excitation spot, as further supported by temperature-dependent IX imaging in panel **b**. At temperatures higher than 50 K, both fast and slow recombination times decrease because of higher non-radiative recombination rates. Error bars correspond to the 95% confidence interval. **d**, PL-weighted average distance plotted as a function of temperature. For temperatures above 150 K, we observe a slight increase in the average transported distance, indicating thermally driven exciton transport^18^. LRID, lattice-reconstruction-induced drift.

Exciton dynamics can be significantly influenced by phonons in the system, making temperature a valuable knob for tuning and investigating the properties of these quasiparticles. Here, we study IX transport and spectroscopic features between 4 K and room temperature. When focusing on the emission from the excitation region, we find very good agreement with previous reports that examined the change of localization regime between localized to delocalized excitonic states, for moiré excitons and free interlayer excitons, respectively^22^. Notably, we find that at liquid helium temperature, moiré excitons dominate the spectra, coinciding with the temperature regime in which we observe fast IX drift transport (Fig. 4b).

It is important to note that the PL signal presented in Fig. 4a is collected from the excitation region and originates from excitons that, rather than propagating rapidly, recombine locally. Interlayer exciton features agree with previously reported moiré-induced localized excitons^22^. In the previous sections, through steady-state and time-integrated measurements, we show that most of the excitons are transported far away from the generation site, while a sizeable portion can still be observed emitting close to the laser excitation spot (Fig. 4b). As the temperature is increased to 80 K, the available thermal energy rises, resulting in a higher rate of tunnelling out of moiré traps, reflected in a comparable population of moiré-trapped and free IXs in the local PL emission. The energy difference between these two peaks, approximately 90 meV, agrees with the estimated exciton barrier height of a moiré potential in R-type WSe_2_/MoSe_2_ (refs. ^18,22,36,37^). However, these two peaks cannot be explained by either singlet and triplet states or by the *K*_CB_−*K*_VB_ and *Λ*_CB_−*K*_VB_ transitions, as the expected energy difference would be limited to 25 meV (refs. ^38–40^) and 55 meV (ref. ^26^), respectively.

As IXs transition from moiré trapped to free excitons, we also observe a gradual quenching of fast exciton transport. Notably, as we increase the temperature to 50 K, the *T*_1_ lifetime increases (Fig. 4c). In the same temperature range, we observe a change in the spatial pattern of the closest recombination site relative to the laser excitation region. Specifically, the distance between the two sites decreases with increasing temperature (Fig. 4b), leading to complete merging of the two recombination sites when the temperature reaches 80 K. Thus, the combined information from the PL imaging and lifetime estimation strongly suggests a drastic decrease in the efficiency of IX drift transport at higher temperatures. In Supplementary Movie 4, we further extend this dataset by measuring density-dependent and temperature-dependent time-integrated interlayer exciton diffusion.

As shown in Fig. 4d, the PL-weighted average distance allows us to visualize a drastic change in the diffusion trend as the system temperature increases. The average distance decreases as the temperature rises to 150 K, in which the remote IX recombination sites are totally deactivated, indicating the absence of fast transport in this temperature range and resulting in a minimum in the average distance. Beyond 150 K and up to room temperature, we observe an increase in time-integrated spatial IX diffusion, which is proportional to the temperature. This behaviour can be explained by thermally activated hopping transport^18^.

Finally, the thermal deactivation of lattice reconstruction-induced remote emitters is evident from IX spatial imaging. At low temperatures, we observe several remote recombination sites with no bright PL emission between them. For temperatures above 150 K, these separate clouds merge into a single bright IX cloud centred around the laser excitation spot. This behaviour is further supported by the full temperature-dependent IX imaging dataset provided in Supplementary Movies 3– 4.

## Conclusion

In summary, we observe a remarkably fast transport of interlayer excitons, which we explain through large excitonic potential landscape variations, induced by lattice reconstruction. This mechanism enables the long-range propagation of excitons with fast drift velocities, efficiently moving a substantial portion of the photogenerated excitons out of the excitation region. Importantly, we find that this transport mechanism remains dominant for temperatures up to 150 K, highlighting its robustness. Our findings underscore the nuanced and complex nature of interlayer exciton transport. Further developments to disentangle the coexisting IX transport mechanisms would require a combination of electro-modulation and pump-probe spectroscopy, enabling the resonant detection of interlayer exciton motion.

Ultimately, our work unveils high mobilities of interlayer excitons in highly inhomogeneous potential landscapes, which opens new possibilities for the development of energy-efficient, high-performance exciton-based integrated circuits. Furthermore, our analysis of the rapid escape of IXs from the excitation region results in the fast dilution of the excited quasiparticle cloud. This phenomenon underscores the importance of assessing exciton density estimates in exciton transport experiments not only within the excitation region, but also in light of the surrounding environment and potential landscape variations. These results have major significance for condensed-matter physics research of density-dependent phase regimes of exciton gases.

## Methods

### Device Fabrication

The full device consists of a Gr/hBN/WSe_2_/MoSe_2_/hBN/Gr heterostructure deposited on a bottom-distributed Bragg reflector mirror (Supplementary Note 1). The Gr gate at the bottom of the stack was fabricated through mechanical exfoliation from bulk graphite (NGS) on distributed Bragg reflector mirrors manufactured by Laseroptik using the ion beam sputtering technique. These mirrors were deposited on Si/SiO_2_ substrates, which are designed to have a reflectivity window at 800–1,000 nm, and consist of 12 pairs of SiO_2_ and Ta_2_O_5_. The heterostructure was fabricated by the dry pick-up^41,42^ transfer technique with a polycarbonate membrane on a polydimethylsiloxane (PDMS) pyramid. Here, layers of WSe_2_ (2D Semiconductors), MoSe_2_ (2D Semiconductors) and hBN were sequentially picked up from the exfoliation substrates. The top hBN layer was first picked up with the polycarbonate membrane from SiO_2_, which was then used to pick up the WSe_2_ and MoSe_2_ layers from Gelpak. Finally, the bottom hBN layer was picked up from SiO_2_. This layered structure was then carefully placed over the bottom Gr layer by gradually increasing the temperature to above 150 °C, ensuring firm adhesion to the substrate. Meanwhile, the top Gr layer was transferred by a two-polymer wet transfer method^43,44^. Here, the bottom polymer was dissolved in a solvent and the top polymer was left free floating to be transferred. After wet transfer, the top polymer layer was cleaned in acetone. Electrical contacts to both the Gr gates and the transition-metal dichalcogenide (TMDC) components within each device were fabricated by electron-beam lithography followed by electron-beam evaporation of metals (2 nm Ti/80 nm Au). The assembled device was then subjected to a 6-h annealing phase at a temperature of 340 °C under a high vacuum environment (10^−5^ mbar). The basic characterization of the device after thermal annealing is provided in Supplementary Note 1.

### Optical Measurements

All optical measurements were performed in a vacuum at 4.6 K, unless stated otherwise, in a helium-flow cryostat. Interlayer excitons were excited using a custom-built confocal microscope, while the emitted photons were collected through the same objective. Optical excitation was achieved with a continuous-wave 640-nm diode laser (PicoQuant, LDH-IB-640-M) focused to the diffraction limit (spot full width at half maximum of 1.2 μm) for steady-state measurements. For micro-PL spectral measurements, the emitted light was filtered by a 700-nm long-pass edge filter and then acquired using a spectrometer (Princeton Instruments SpectraPro 500) and recorded with a charge-coupled device camera (Princeton Instruments, Blaze 400-HR/HRX). Spatial imaging of the IX emission was performed using a charge-coupled device camera (Andor Ixon) with an 850-nm long-pass edge filter that removed both the laser line and the intralayer emission from WSe_2_ and MoSe_2_. For time-resolved measurements, a supercontinuum white laser (Fianium FIU-15) was used, achieving pulse widths smaller than 50 ps at a repetition rate of 1 MHz. The collected photons were spatially filtered with our galvo-scanning system and coupled into a multimode optical fibre. The fibre was then connected to an avalanche photodiode (MPD, Photon Detection Module). The output of the avalanche photodiode was connected to a time-correlated photon-counting module with a resolution of 12 ps r.m.s. (PicoQuant, PicoHarp 300), which measured the arrival time of each photon. We set the time bin to 16 ps or 4 ps for the measurements presented in this work for full decay or rise time estimation, respectively. The single-photon timing resolution of the avalanche photodiode is ~50 ps, which is the main time limitation for this set-up. The technical details can be found in Supplementary Note 11. The average distance (AD), which describes the distance IXs travel from the laser excitation spot to the recombination sites, is defined as follows:

1 $$\mathrm{AD}(T)=\frac{\sum _{x,y}\mathrm{PL}(x,y,T)\times \sqrt{{\left(x-{x}_{\mathrm{laser}}\right)}^{2}+{\left(\,y-{y}_{\mathrm{laser}}\right)}^{2}}}{\sum _{x,y}\mathrm{PL}(x,y,T\,)}$$

where PL(*x*,*y*,*T*) is the temperature and spatially dependent PL intensity, *x* and *y* are the coordinates over which the sum is considered, and *x*_laser_ and *y*_laser_ are the laser centre coordinates.

## Online content

Any methods, additional references, Nature Portfolio reporting summaries, source data, extended data, supplementary information, acknowledgements, peer review information; details of author contributions and competing interests; and statements of data and code availability are available at https://doi.org/10.1038/s41563-026-02688-2.

## Supplementary information

Supplementary Information Supplementary Notes 1–11, Supplementary Figs. 1–18.

Supplementary Video 1 Hyperspectral spatial PL map shown as a function of decreasing photoluminescence energy.

Supplementary Video 2 PL imaging as a function of excitation laser power.

Supplementary Video 3 Temperature-dependent PL imaging.

Supplementary Video 4 Temperature and laser excitation power-dependent PL imaging.

## Data Availability

The data that support the findings of this study are available on Zenodo at https://doi.org/10.5281/zenodo.20697368 (ref. ^45^).
