# Supplementary material for: Fast interlayer exciton drift driven by lattice reconstruction in a van der Waals heterobilayer
Source: Nat Mater. 2026 Jul 29;25(11):1968–74. doi: 10.1038/s41563-026-02688-2 (PMC13751306; doi:10.1038/s41563-026-02688-2)
Supplement: Supplementary file 1 [file 41563_2026_2688_MOESM1_ESM.pdf]

nature materials

Supplementary information https://doi.org/10.1038/s41563-026-02688-2

Fast interlayer exciton drift driven by lattice reconstruction in a van der Waals heterobilayer

In the format provided by the authors and unedited

1. Basic device characterization 2
2. IX dipole moment estimation 3
3. Steady-state power-dependent IX imaging 4
4. FDTD Simulation of vdW heterostructure 6
5. Time-resolved photoluminescence fitting 8
6. Drift velocity estimation 10
7. Generated interlayer exciton density estimation 11
8. Interlayer exciton energy characterization and study 12
9. FEM Simulation of IX Propagation 14
10. Power-dependent IX transport 17
11. Experimental setup 19
References 21

1. Basic device characterization

Figure S1a shows the optical microscopy image of the device under study. Optimal interlayer coupling is confirmed by room temperature widefield PL as shown in Figure S1b. The absence of large bubbles is both confirmed by optical (Figure S1a) and atomic force microscopy (Figure S1c), except for a few nanometric bubbles within the heterostructure. This exceptional flatness, in spite of the complex structure, allows us to neglect any effect of morphological bubbles on the IX exciton transport dynamics, and its unique flatness limits our characterization to this specific sample. Furthermore, employing the DBR mirror as substrate for the heterostructure enables us to further improve collection efficiency of the PL signal.

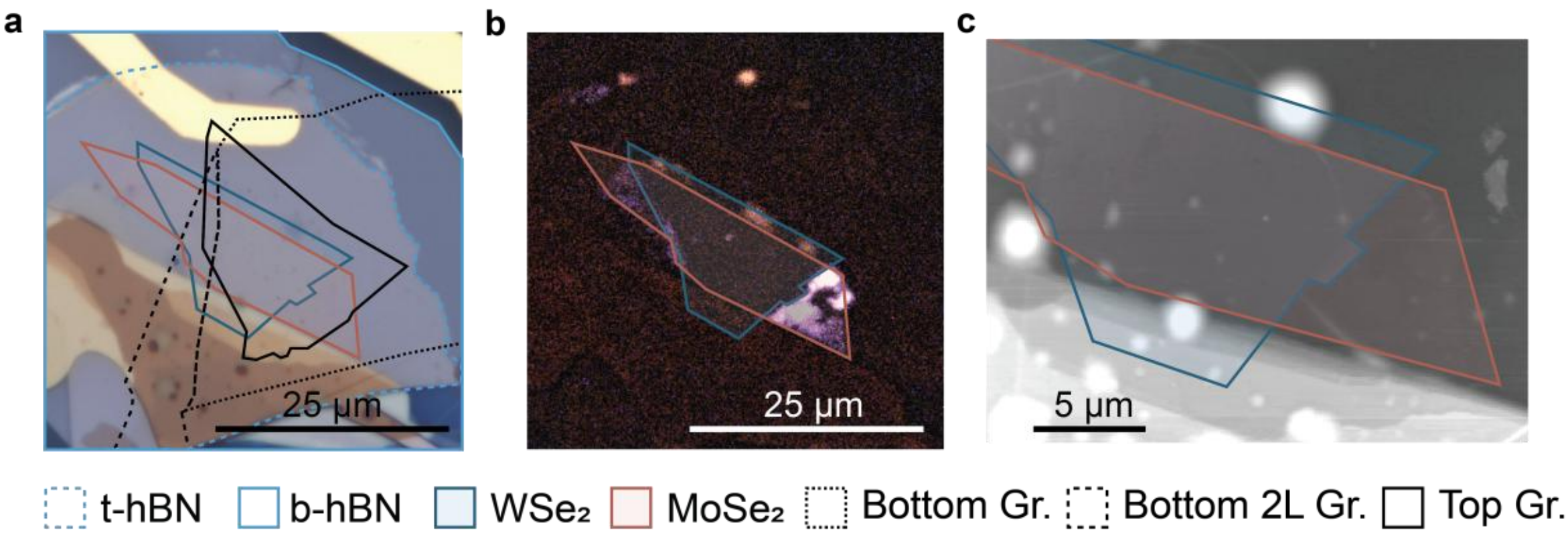

Figure S1 – Device outline. a, brightfield optical microscopy image. The shapes of the relevant flakes are overlayed as indicated by the legend. **b,** Dark-field widefield PL image showing bright PL for the $MoSe_2$ flake, and few bright spots of WSe_2. The overlap region shows fully quenched PL for both monolayers, as an indication of charge transfer. **c**, Atomic force microscopy image of the heterostructure before top graphene transfer. The AFM scan shows minimal presence of morphological bubbles, confirming atomically smooth interfaces between the TMDCs monolayers.

We proceed by investigating the stacking order and twist angle of the TMDC monolayers by second harmonic generation (SHG), which has been conducted at room temperature in a high vacuum environment. Figure S2a shows the spatial map of the emitted SHG signal in log scale. A clear intensity enhancement is observed within the heterobilayer region, which has been identified as a fingerprint of R-type stacking order[1]. TMDCs crystal lattice features a 3-fold rotation symmetry. The SHG signal is largest if the excitation polarization is parallel to one of the crystalline axes. Thus, the emitted SHG signal follows[1]:

$$I_{SHG} \propto \cos^2(3\theta - 3\varphi_{TMDC}) \tag{1}$$

with θ being the laser polarization angle, and φ_{TMDC} the crystalline axis orientation. The polarization dependence of the SHG signal allows to recover the crystalline axis orientation for both TMDCs (Figure S2b), and to then estimate their twist angle. In this sample, we obtained $\theta_{twist} \sim 3.35° \pm 0.71°$.

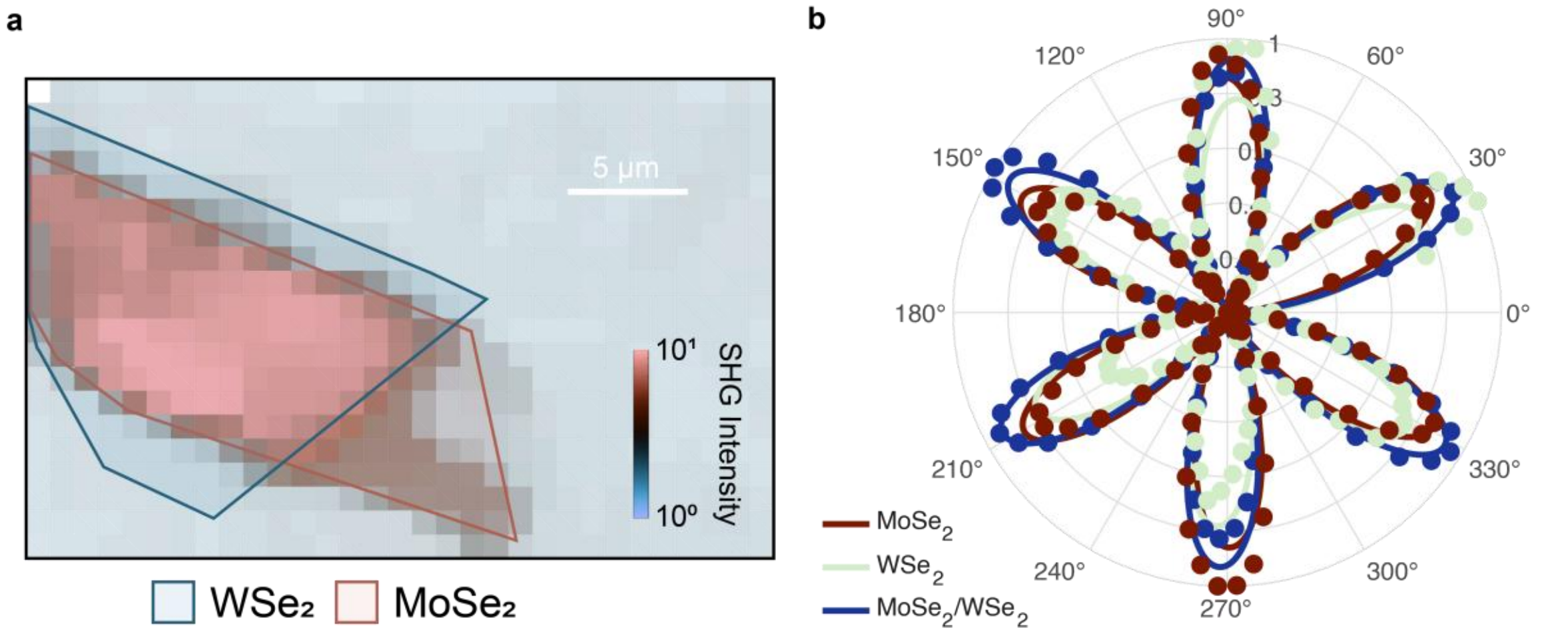

Figure S2 -Twist angle identification by SHG. a, SHG spatial mapping in logarithmic color scale. The intensity of the overlap region is several times larger than SHG intensity from monolayer $MoSe_2$, confirming R-Type stacking order. **b**, polarization-dependent SHG measurement of the TMDCs monolayers and heterostructure region (scatter points) with respective sinusoidal fits (solid lines). Sinusoidal fits enable to estimate a twist angle between monolayers of $\theta_{twist} \sim 3.4° \pm 0.7°$.

We conduct basic device characterization of the device under study by PL spatial mapping at cryogenic temperatures. Optimal coupling between the TMDC monolayers is confirmed by the quenched intralayer PL emission of $MoSe_2$ shown in Figure S3b. The large intensity variation of the emitted IX PL is observed within the heterostructure region (Figure S3a) likely induced by lattice reconstruction disorder, as further discussed in Supplementary Note 4. We provide the laser reflectance spatial mapping in Figure S3c as a reference.

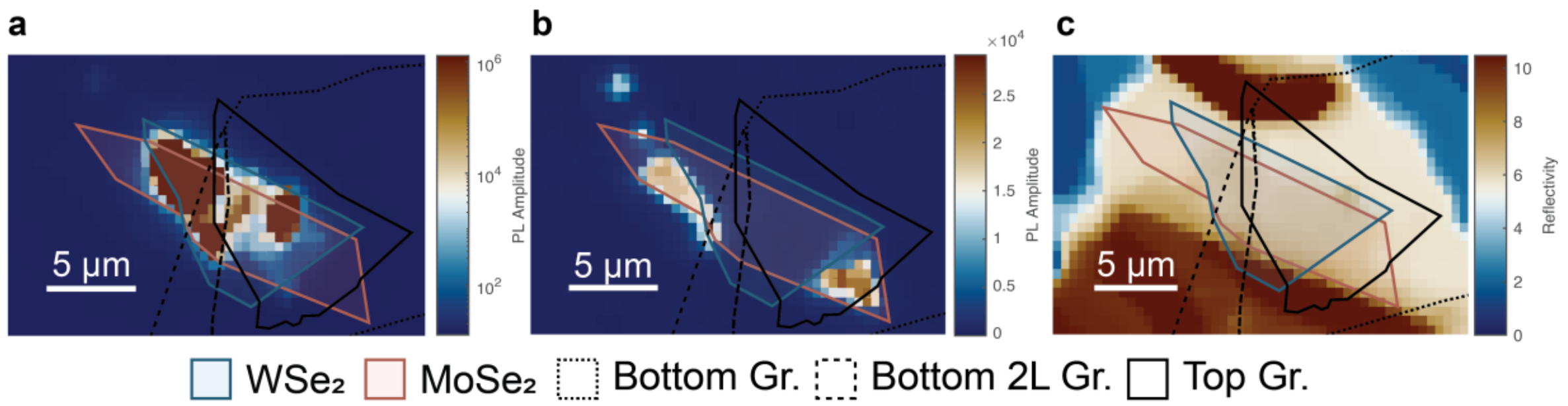

Figure S3 - Spatial PL mapping. a, Interlayer exciton PL map performed by laser scanning across heterostructure area. Large PL intensity variation is observed due to mesoscopic lattice reconstruction. **b,** $MoSe_2$ intralayer PL emission, showing full quenching in the heterobilayer region. **c**, Laser reflectance map provided as reference.

2. IX dipole moment estimation

We prove the out-of-plane dipolar nature of our interlayer excitons by the quantum-confined Stark effect, as shown in Figure S4a. In particular, we recorded the field-dependent IX PL obtained by exciting the structure with a low continuous-wave (CW) laser excitation power of 1.8 μW. To apply a vertical electric field without inducing any electrostatic gating, we applied top and bottom gate voltages following the relationship $V_T = -\alpha V_B$, where $\alpha = \frac{t_{t-hBN}}{t_{b-hBN}}$ denotes the ratio of the hBN layer thicknesses at the top and bottom (28 nm and 31 nm respectively).

The electric field was then calculated using the equation:

$$E_z = \frac{V_B - V_T}{d_{tot}} \frac{\epsilon_{hBN}}{\epsilon_{hb}} \quad (2)$$

Here, $\epsilon_{hb} \approx 7.5$ represents the dielectric permittivity of the $WSe_2/MoSe_2$ heterobilayer region, $\epsilon_{hBN} \approx 4$ represents the dielectric permittivity of hBN, and $d_{tot} = d_{hBN}^{top} + d_{hBN}^{bot} + d_{WSe2} + d_{MoSe2}$, with $d_{WSe2} + d_{MoSe2} \sim 1.3\ nm$, $d_{hBN}^{top} \sim 28\ nm$, and $d_{hBN}^{bot} \sim 30\ nm$. The IX dipole length, extracted from the Stark shift depicted in Figure S4a, is estimated *via*:

$$d = -\frac{1}{e} \frac{\partial \Delta E_{IX}(E_z)}{\partial E_z} \quad (3)$$

where $\frac{\partial \Delta E_{IX}(E_z)}{\partial E_z}$ is the energy shift of the dominant IX peak with the applied field. This estimation yielded a dipole length (shown in Figure S4b) of 0.5 nm, consistent with previous studies on interlayer excitons in $WSe_2/MoSe_2$ heterobilayers[2–4].

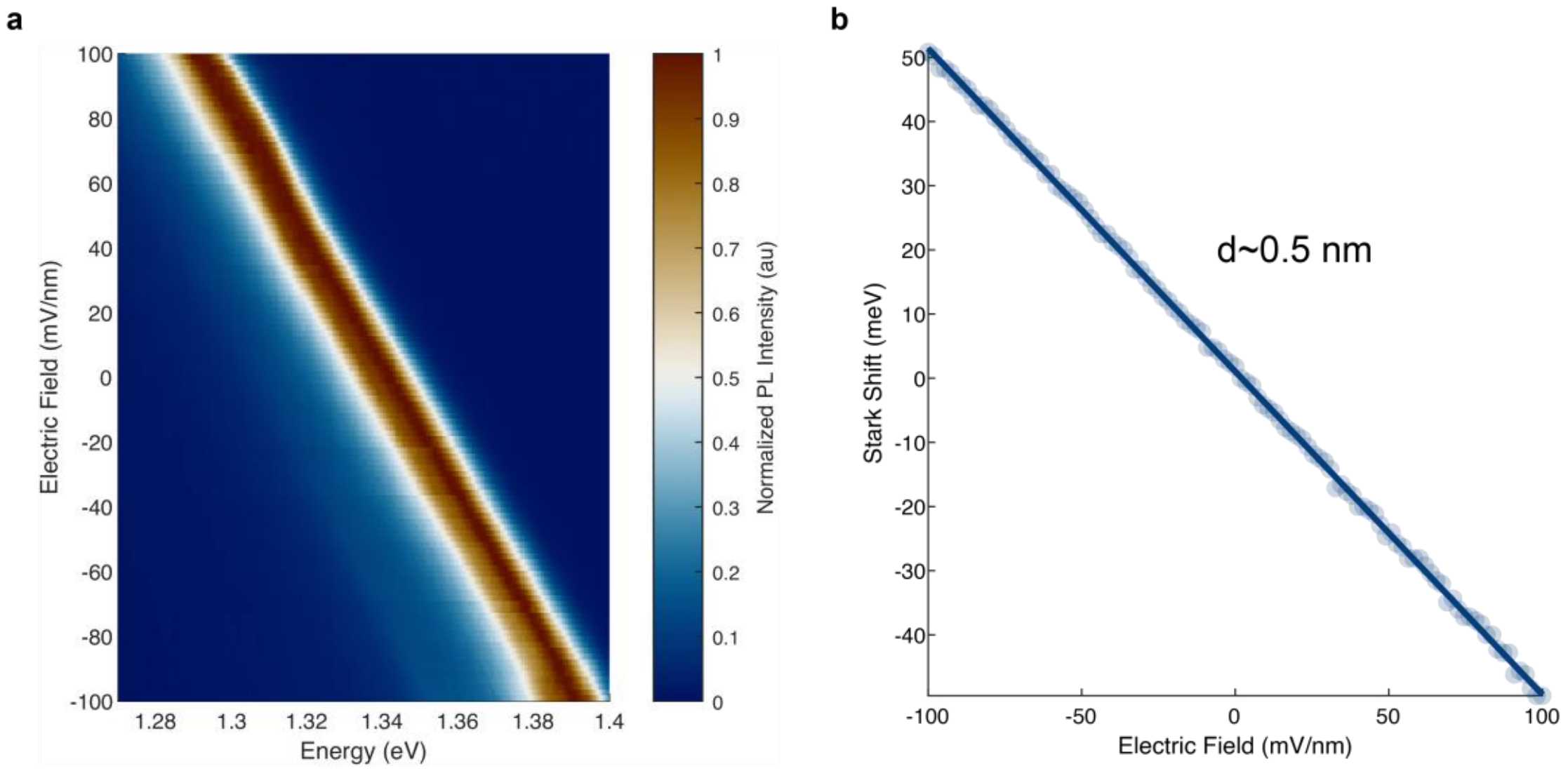

Figure S4 - Quantum-confined stark effect measurement. **a**, Stark effect map at low excitation power, 1.8 µW. **c**, Stark shift estimation performed extracting the energy of the maximum PL intensity as a function of applied vertical electric field.

3. Steady-state power-dependent IX imaging

Once the interlayer nature of the excitonic species under study has been confirmed, we can shift our focus on imaging their spatial diffusion. The highly inhomogeneous emission patterns obtained by CW PL imaging are shown in Figures S5a,c. This pattern exhibits several recombination spots, with a maximum distance from the laser excitation spot of up to 15 µm according to the specific laser spot location. Notably, the spatial PL emission pattern is mostly unperturbed by changes in the laser location (highlighted by a large red circle in Figure S5a,c).

Nevertheless, the relative emission intensities of the emission sites are significantly affected by the laser position.

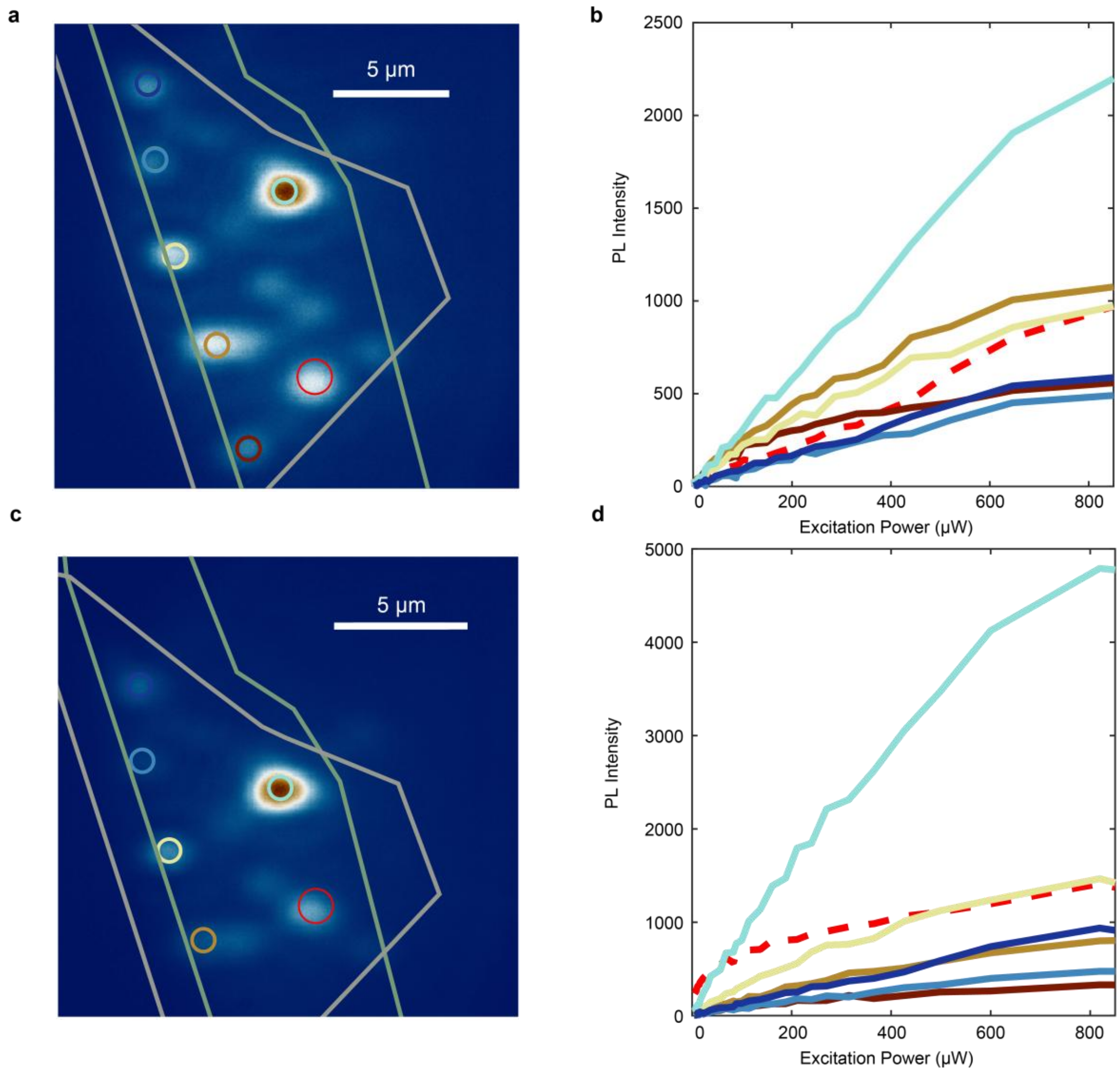

Figure S5 - CW power-dependent IX diffusion. a,c, CW IX PL imaging obtained at large excitation power (≈900µW) applied within the large red circle at different positions within the heterostructure. Colored circles show the position of the integrated PL intensity plotted in b and d respectively. **b,d**, spatially integrated PL intensity plotted as a function of optical excitation power. Red dashed line shows the integrated PL intensity from the laser spatial position.

We analyzed the integrated emitted PL as a function of laser excitation power (Figure S5b,d). The PL intensities in Figure S5b (d) are extracted from the areas of the corresponding color-matched circles in Figure S5a (c). The red dashed line in Figure S5b,d indicates the PL intensity emitted from the same area where the laser excitation is applied. The power-dependent behavior of the integrated PL intensity offers crucial insights into the type of transport phenomena. In particular, the PL emitted from recombination sites far from the excitation spot is often larger than the PL emitted from the excitation area (Figure S5b,d). Furthermore, the relative PL intensity among the different recombination sites changes significantly with the optical excitation power and the specific laser excitation position. In Figure S5d, we show how the PL emitted from the laser excitation spot is the highest at low excitation powers but is rapidly overcome by the PL emitted from remote recombination sites as the optical excitation

power increases. This shows how IX interactions can significantly affect the long-range transport observed in this study. In Figure S6a-d we plot the CW IX PL imaging at different excitation powers. Strikingly, long-range transport is present even at low excitation power (Figure S6a), although the maximum PL intensity is located close to the laser excitation spot. As the power is increased the relative intensity between the laser excitation and remote recombination sites is drastically changed, featuring larger intensities in remote recombination sites. This is of fundamental importance as it shows that long-range transport is present even for low CW excitation powers ($P_{exc} < 1\ \mu W$) corresponding to exciton density regimes below the Mott transition ($n_{IX} < 10^{12}\ cm^{-2}$).

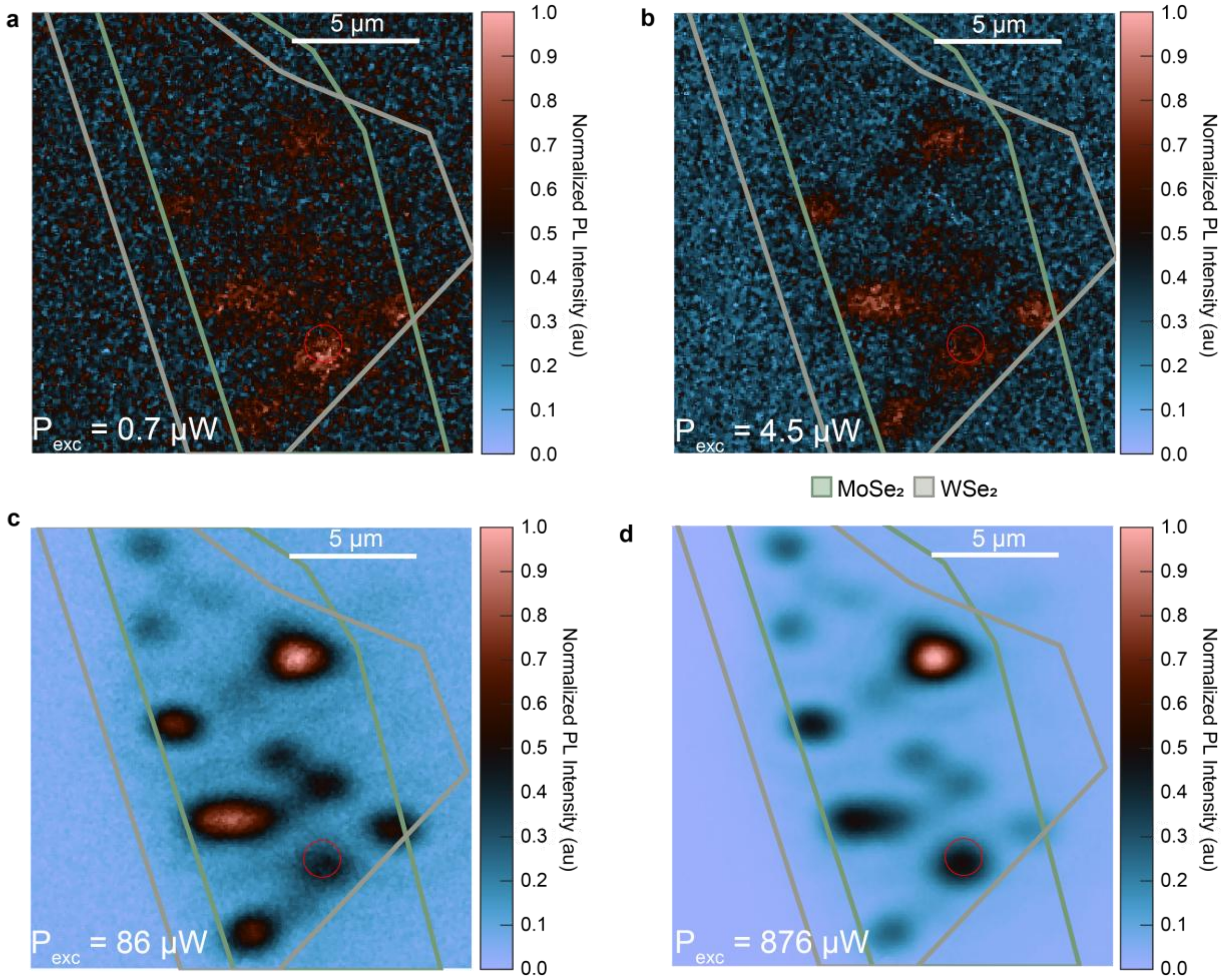

Figure S6 - Power dependent CW IX PL imaging. **a-d**, CW IX PL imaging at 0.7 µW(a), 4.5 µW(b), 86 µW(c) and 876 µW(d). Long-range transport is confirmed at low optical excitation power, which corresponds to generated exciton densities below 10^{12} cm^{-2}. On the other hand relative PL intensity of the recombination sites changes drastically with excitation power, evidencing the contribution of density-dependent transport mechanisms, such as repulsive interactions and Auger scattering, in the long-range transport.

4. FDTD Simulation of vdW heterostructure

Such a peculiar spatial pattern can be well explained by large lattice reconstruction disorder, which is significant in mechanically stacked van der Waals heterostructures. S. Zhao recently correlated large variations of the lattice reconstruction pattern in H- and R-type stacking heterobilayers of $WSe_2/MoSe_2$ with their PL emission characteristics[5]. They observed a large 2D reconstruction pattern arising from the sample edges and other non-idealities, e.g., bubbles. These 2D domains would first mesh into 1D line domains and after in 0D confined domains as the distance from the edges or bubbles increases. In agreement with their findings for R-type

stacked heterobilayers, we observe bright PL emission from 2D reconstructed domains, which arise at the heterostructure edge and close to non-idealities, with a mixture of bright and dark states in 1D domains and further reduced emission from 0D domains as the distance from the edge increases[5].

It is then clear that the observed spatial PL pattern does not agree with the spatial emission pattern expected from waveguided modes, which are expected to exponentially decrease with the distance from the laser excitation area[6]. By contrast, larger emitted PL intensities from recombination sites far from the laser excitation, suggest highly effective exciton depletion from the photogeneration region. This is further corroborated by time-resolved analysis in the next section. Nevertheless, to further exclude the possibility of laser light waveguided to remote sites and excite IX PL, as a driving force for the studied transport dynamics, we investigate the amount of waveguided excitation light in our heterostructures. This is performed by conducting Finite-Difference Time-Domain (FDTD) simulations with Tidy3D solver. A concise overview of the performed simulations is presented in Figure S7, where we analyze and compare the mode propagation of the first TE mode at 680 nm. We carry out this simulations on two different structures, the first being equal to the one used in this work, and a second one where a total 200 nm thick hBN stack is considered for comparison. In fact, waveguiding of light in this structure is expected to lead to significant effects only for hBN stack thickness larger than 100nm[7]. Figure S7a depicts the simulated device in the bottom panel (substrate, DBR and heterostructure) along with the field monitor (yellow panel) and the injection point (slightly inside the structure, so to ensure coupled mode). Due to structural symmetry, periodic boundary conditions are imposed in the z-direction, whilst perfectly-matched-layers (PML) boundary conditions are chosen in the x-y-directions. In order to avoid highly computational costs, the TMDCs and the Gr flakes are defined as 2D mediums. While we are aware that in our experiment, only scattered light might couple to in plane modes and excite IX PL in remote sites, we carry out our simulations with a mode source propagating in the positive y-direction to investigate the possibility of mode propagation. Specifically, we focus on the TE_0 mode which is expected to propagate for thicknesses lower than 100 nm at wavelengths around 680 nm[7].

Simulations show a direct propagation of injected mode in a bare hBN sample with large vdW stack thickness (i.e. 200 nm), as demonstrated by the oscillating field in Figure S7c, in accordance with ref.[7]. By contrast, we expect no mode propagation in the devices investigated throughout this work, (i.e. total vdW stack height roughly equal to 60nm). In fact, in this case the electric field intensity is damped by 3 orders of magnitude with respect to the injected intensity, as shown in Figure S7d.

We also point out, that we expect laser light waveguided in our structure, to lead to even lower contribution in the PL excitation. In fact, we excite PL in a confocal microscope, with out-of-plane incident excitation, therefore the total waveguided light would be less than 3 orders of magnitude of the in-plane scattered light at the excitation spot, leading to an even smaller contribution, and therefore negligible. Finally, in our simulations we don't account for any light reabsorption effect of the IX PL, as IX feature drastically low oscillator strengths, therefore leading to negligible contribution of this effect. In conclusion, the performed FDTD study combined with the observed PL trend as a function of distance allows us to rule out the waveguiding effect as an explanation for the observed remote exciton recombination.

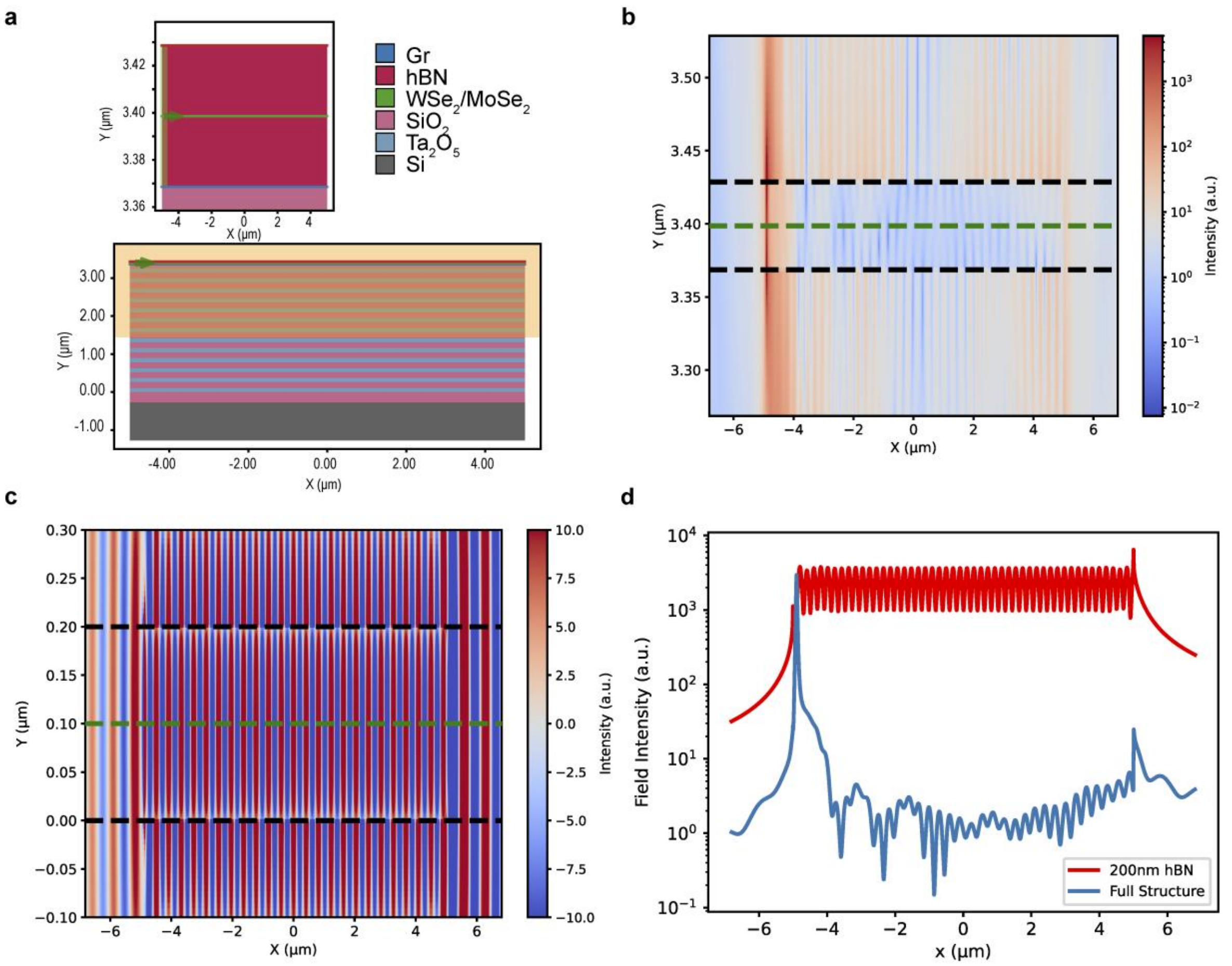

Figure S7 - FDTD waveguiding simulation. **a**, Cross section of the simulated structure at z = 0 µm. The bottom panel illustrate the full simulated structure (in yellow is shown the field monitor). The top one is a zoom highlighting the 2D heterostructure. The green arrow represents the injected TE mode at 680 nm. **b**, Spatial distribution of the electric field intensity of the mode source in the zoomed in view of panel a. The green dotted line represent the TMDCs position, whilst the black ones the Gr gates. **c,** Real part of the electric field distribution y-component for a 200 nm thick hBN flake, oscillating field is observable as a proof of mode propagation. **d,** Comparison of the injected mode propagation in the aforementioned cases: electric field intensity linecut at the TMDCs position in **b** and at the center of the 200 nm hBN flake in **c.**

5. Time-resolved photoluminescence fitting

To analytically estimate the speed at which IXs travel to the observed recombination sites, we estimate the rise rate of the TRPL signal from every site referenced to the rise rate estimated from the TRPL signal collected at the laser excitation region. In this work, due to comparable system time resolution and magnitude of the observables, combined with the absence of an a priori knowledge of the transport model, we employ a probabilistic fitting approach. This methodology circumvents the need for a model describing the rise and subsequential decay models, by adopting a cost-reduction optimization algorithm that finds the optimal probability distribution for a logarithmic distribution of decay and rise rates. For this estimation, we employed the previously reported implementation[8], exploiting a basic-pursuit denoising (BPDN) optimization algorithm[9]. This choice, among other maximum entropy-based methods, was limited by the need to model both rise and decay rates, represented by negative and positive probability distributions respectively. The BPDN is indeed the only optimization algorithm readily available that supports optimizations of weights of both polarities[8].

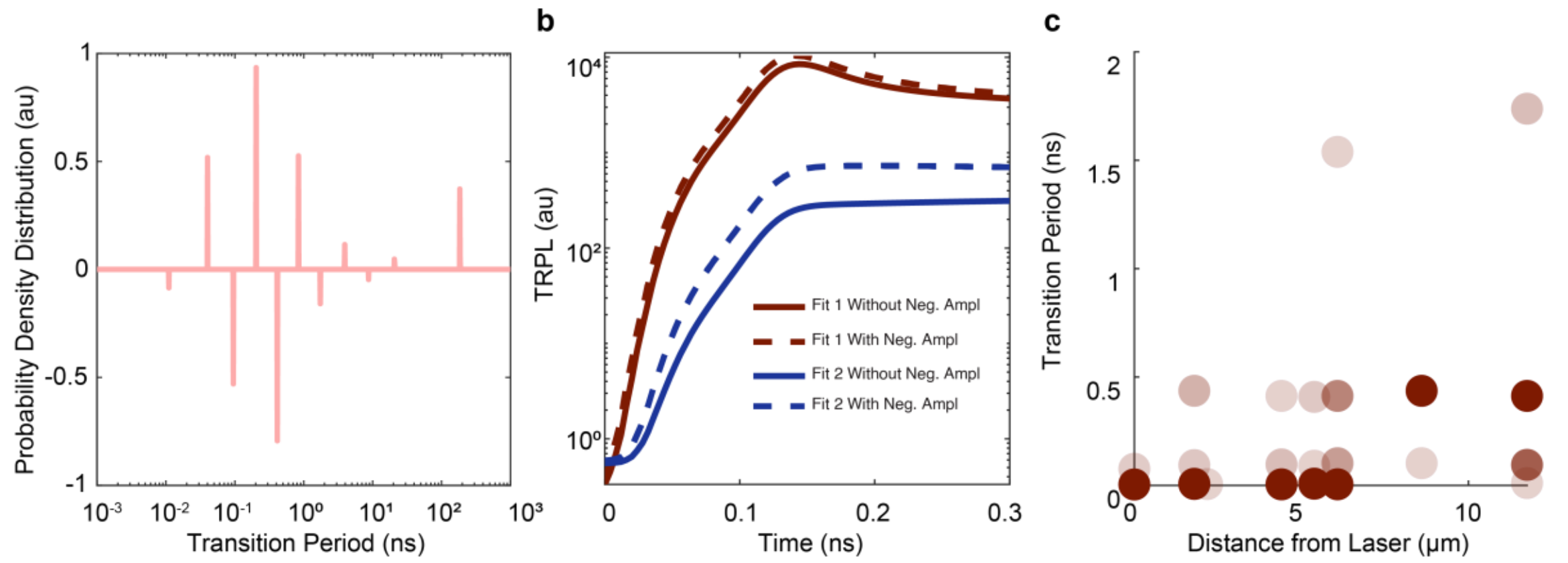

Figure S8 - TRPL fitting procedure. **a**, The probability distribution of the rise and decay times optimized to fit the recorded TRPL signal. Negative (positive) probabilities represent the absolute values of the probabilities for decay (rise) times. **b**, Evaluated model with fitted probability distribution with (dashed) and without (solid) negative probabilities. Evaluated models for TRPL emitted from the laser (farthest) region are plotted in red (blue). In agreement with expectations, the TRPL signal from the laser (farthest) region shows shorter (longer) rise times. **c**, Scatter points summarizing the negative probability distribution fitted with respect to the distance from the optical excitation spot. The transparency of each scatter point is normalized at every distance with respect to the largest probability.

In Figure S8a we show a typical probability distribution function for a logarithmically spaced array of rates after fitting to one of our datasets. We further confirm the rise rate nature of the negative probabilities obtained from the fitting algorithm, by evaluating the fitted function for the data collected at maximum (blue) and minimum distance (red) in Figure S8b with only positive (solid), as well as both positive and negative (dashed) probabilities. This clearly shows a faster rise of the TRPL signal for the evaluation that includes probabilities with both signs. Notably, the rise signal evaluated with only positive probabilities is induced by the convolution of the fit with the experimental instrument response function. This step is necessary to decouple observables with comparable magnitudes with respect to the system time resolution. Once the fitting has been performed for all the acquired datasets, we plot the rates of the negative probabilities, as we are only interested in the rise dynamics, with respect to their respective distance from the laser excitation (Figure S8c). We note that the transparency of the scatter points has been mapped to their relative probability normalized for every distance.

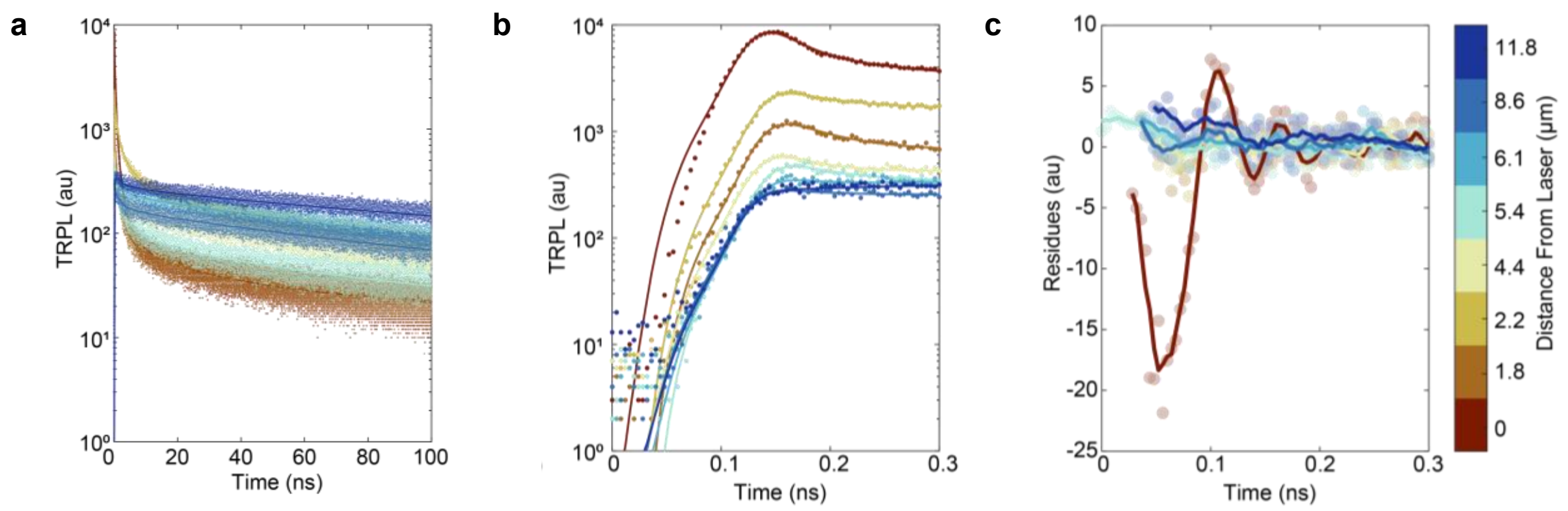

Figure S9. Time resolved PL fits. **a**, Fit (solid line), and TRPL signal (scatter points) showing excellent agreement in the intermediate time range. **b**, Zoom in for short time range of TRPL (scatter data) and fits (solid line). **c**, residuals of the fit for TRPL signal collected at every recombination spot. The color bar shows the distance of the collected signal from the laser excitation spot for all the panels.

We further show the high-quality of the fitting results in Figure S9. Excellent agreement is found for the fit performed (solid lines) and the acquired TRPL data points (scatter points) at both long (Figure S9a) and short (Figure S9b) time scales. The fitted residuals show variations within less than 10 TRPL counts for all the data points, with the only exception being for the data point acquired in the laser excitation spot. The deviation of this dataset is most likely induced by the fast rise time beyond our system resolution. Nonetheless, this specific estimation will be used as a time reference for other rise times, thus the induced rigid shift will be reflected equivalently on the other estimation. The uncertainty resulting from the discrepancy between the obtained time-resolved signal and the fitted model does not significantly impact the subsequent velocity estimation process.

Due to the computational demand of the BDPN algorithm to extract the aforementioned rise rates, it is unfeasible to use the same algorithm for the estimation of the IX lifetime on the full time range needed to estimate the full IX decay. For this purpose, we employ a simpler double exponential decay model, whose fit results are shown in Figure S10. Interestingly, we observe a large decrease of the imbalance between the fast and slow decays as the distance is increased from the laser excitation region. This qualitatively agrees with the hypothesis that the observed fast decay is a consequence of the fast depopulation from the laser excitation region towards the remote recombination sites.

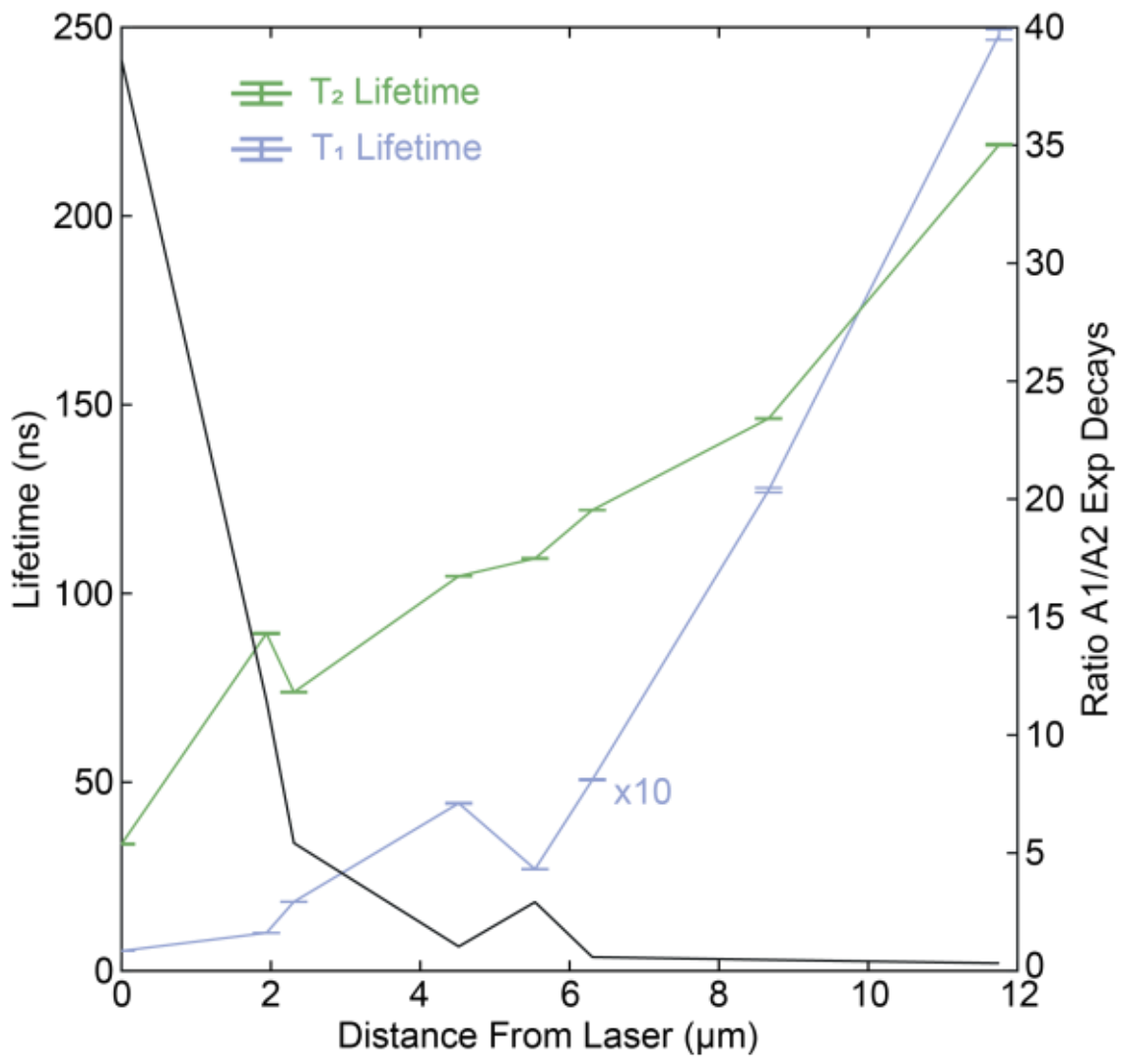

Figure S10 – Decay lifetime estimation. The TRPL signal is modeled with a double decay exponential decay, and the two estimated decay lifetimes T_1 and T_2 are shown in green and blue respectively. On the right axis the ration between the fitted amplitudes of the two decays is plot. Error bars correspond to standard deviation around the mean.

6. Drift velocity estimation

In our work, we spatially sample the recombination sites and correlate their distance from the laser position with the TRPL signal. Therefore, we employ the mean square displacement of the exciton cloud given by $\langle x^2 \rangle(t)$. In Figure 2c in the main text we plot the squared distance from the laser excitation with respect to the estimated average rise time as previously described.

This is modeled with a nonlinear trend highlighting the necessity to consider a drift-diffusion model in order to fit the experimental data[12–14]:

$$\langle x^2\rangle(t) = \langle x^2\rangle(0) + 4D_0 t + (v_d t)^2 \quad (4)$$

where $\langle x^2\rangle(0)$ is obtained considering the laser half-width half-maximum of 0.65 µm. Moreover, D_0 is the intrinsic diffusivity coefficient, $v_d = \mu_f E_f$ is the drift velocity, μ_f is the field-effect mobility and E_f is the effective field driving IX motion extrapolated from the quasi-linear variation of the potential from Figure 2. In this equation, we are taking into account the fact that the averaged squared distance is a two-dimensional distance.

We note that other works have resorted to employing an effective diffusivity term to encapsulate second-order terms dependent on the excitonic density (i.e. exciton-exciton annihilation, or energy renormalization terms given by interactions)[15–20]. While our simplified drift-diffusion approach does not explicitly consider second order terms, we further delve into the role of interactions in our measurements within Supplementary Note 3 for completeness.

7. Generated interlayer exciton density estimation

In the main text, we discuss time-resolved IX transport with varying exciton density regimes. In this supplementary note, we show how we conduct the estimation of the generated exciton density. First, we establish the relationship between generated exciton density and the excitation laser powers employed in this study. In our time-resolved characterization, we utilized as an excitation source a supercontinuum white laser with a 1MHz repetition rate, with a central wavelength at 680 nm and bandwidth of approximately 10nm. We estimate the generated exciton density under pulsed excitation as[15,21]:

$$\mathrm{n}_0 = \frac{P \cdot \alpha}{A \cdot f_{rep} E} \quad (5)$$

with P being the average laser power in Watts, A the laser excitation area in cm^{-2}, f_{rep} the repetition rate in MHz, α is the absorption coefficient and E is the photon energy in eV. The spatially resolved absorption coefficient in our heterostructure has been estimated using a 4x4 transfer matrix formalism[22], adapting the reported algorithm implementation[22,23] for our heterostructure geometry. Transfer matrix simulations were performed using refractive indexes reported in [24–26] for hBN, WSe_2/$MoSe_2$, and graphene respectively. In Figure S11a we plot the computed absorption coefficient for both TMDC layers. The featured large modulation is inherited by the DBR reflectivity, whose reflectivity window is centered around 1.4 eV. We compute the average absorption coefficient taking into account the laser spectra excitation by:

$$\alpha = \frac{\sum_{E=1.75eV}^{1.9eV} I(E) \cdot \left(\alpha_{MoSe_2}(E) + \alpha_{WSe_2}(E)\right)}{A \cdot f_{rep} \cdot E} \quad (6)$$

where $I(E)$ is spectrally resolved normalized laser intensity, α_{MoSe_2} and α_{WSe_2} are the partial absorption coefficients in their respective layers. In Figure S11b we show a detailed view of the absorbed light portion in each layer with the spectrally resolved laser intensity. Furthermore, we use the normalization of the absorption coefficient computed from the carrier-density-dependent solution of the semiconductor Bloch equation (SBE), in order to account for the absorption saturation effect as a function of the optical excitation power[21]. This enables the estimation of the power-dependent generated exciton density as shown in Figure S11c.

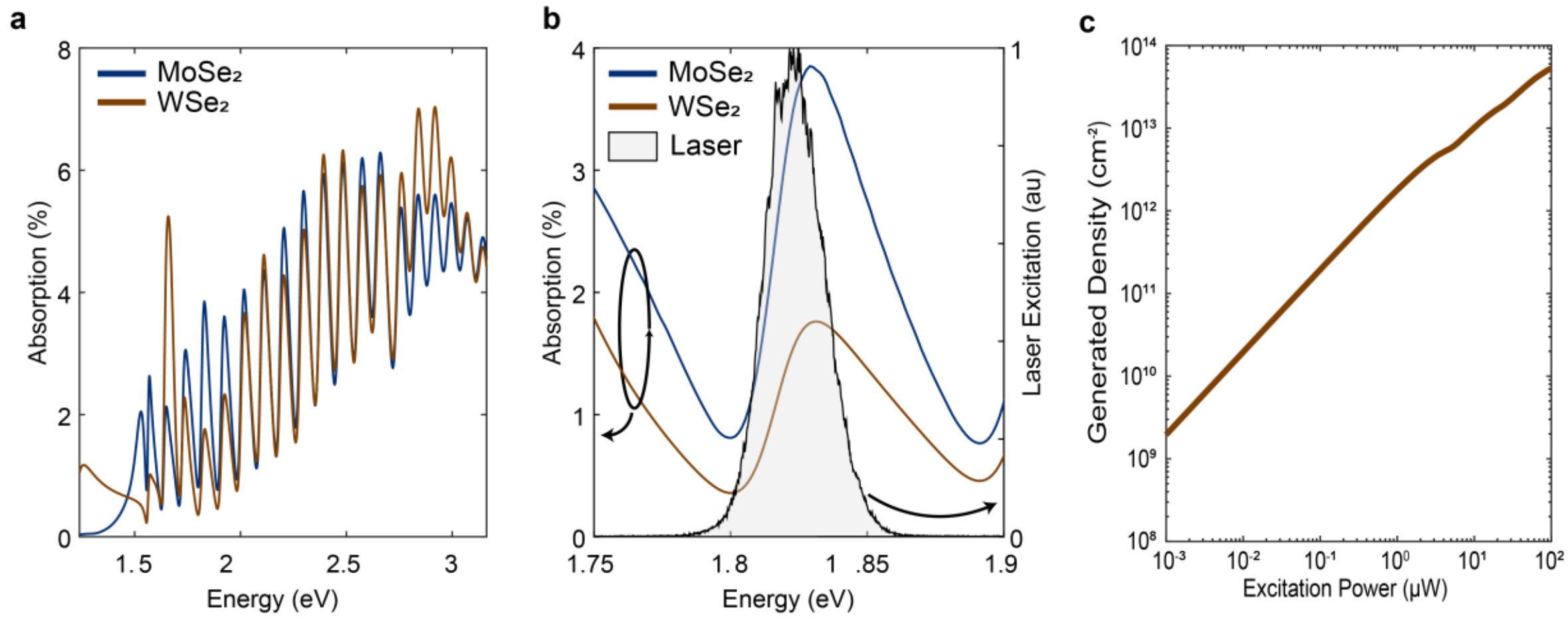

Figure S11 - Estimated absorption and generated exciton density. **a**, Estimated absorption in WSe_2 and $MoSe_2$ monolayers by transfer matrix method implemented in a generalized 4x4 formalism. **b**, Close-in view of the absorption coefficient of each layer, and spectrally resolved excitation laser used to optically generate interlayer excitons. **c**, estimated generated IX density as a function of optical excitation power considering both carrier density absorption renormalization, as previously reported[21], and estimated absorption in our heterostructure geometry by transfer matrix simulations.

8. Interlayer exciton energy characterization and study

First, in order to understand the behavior of interlayer exciton in lattice reconstructed inhomogeneous potential landscapes, we study the time and energy features of interlayer excitons recombining in the excitation region. We perform confocal streak camera measurements of the interlayer exciton emission, whose light first passes through a spectrometer, which is optically coupled to a streak tube camera system. This, allows us to recover both time and energy resolution from the emitted PL. In Fig. S12a we present the emitted PL intensity plotted in the time and energy domain. This clearly shows a change in the most prominent emission peak within the first few tens of picoseconds. To better resolve this dynamic change in emission energy, we fit the emission spectra at each time frame, we show a subsample of these fits in Fig. S12b. The emitted spectra are well described with a double gaussian peak model, which enables us to extract the peaks energies as a function of time. This is summarized in Fig. S12c for the entire dataset. We note that we plot the scatter points in Fig. S12c by normalizing their size relative to the weighted amplitude of their respective gaussian peaks. This provides an easy way to visualize the most prominent contributions to the emitted

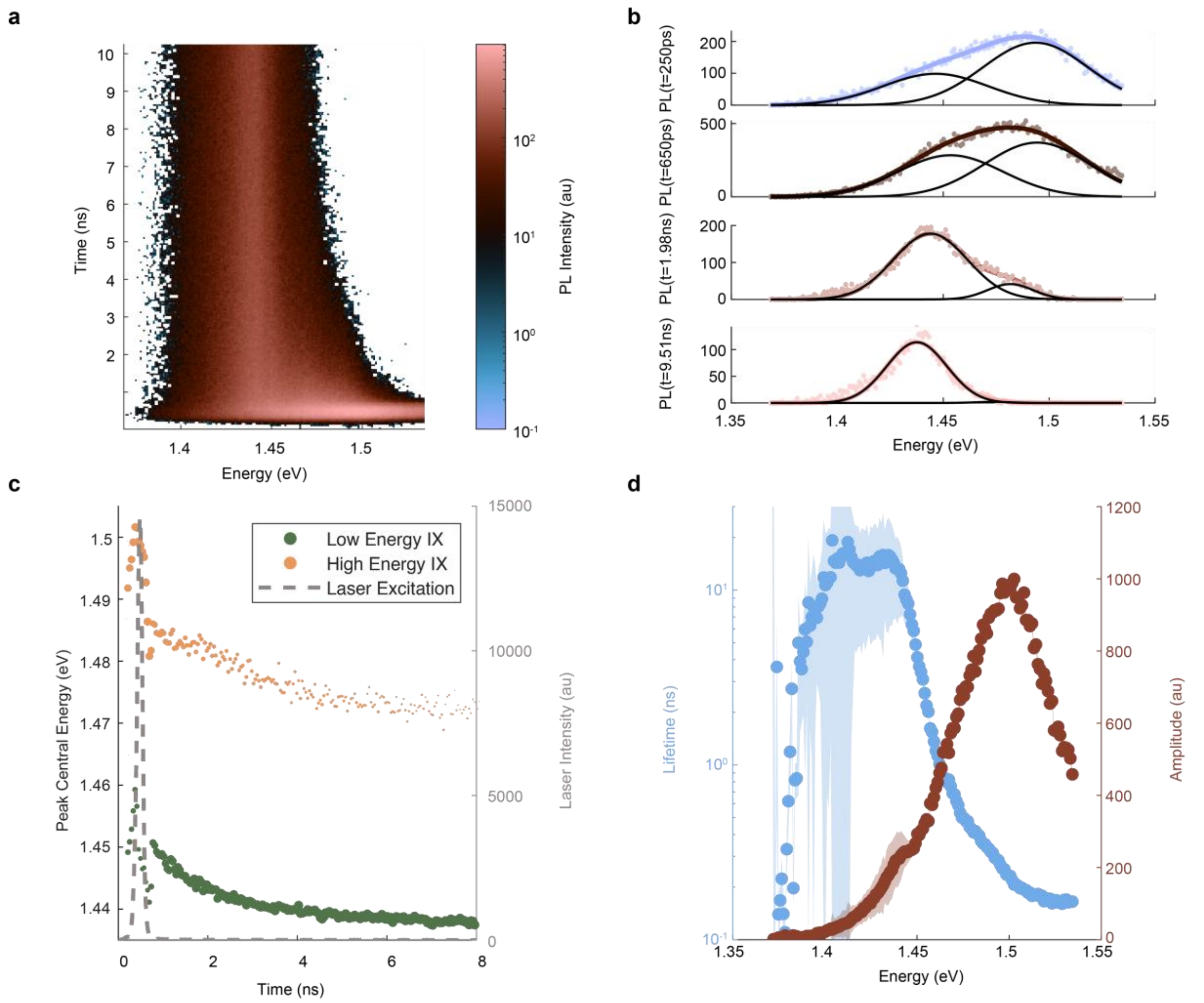

Figure S12 – Energy-time resolved IX PL emission. **a**, Streak camera measurement of IX. **b,** PL spectra extracted from streak camera measurement. Black thin lines represent two gaussian peaks that have been fit to the total PL signal at each time. **c,** Energy of the fitted gaussian peaks as a function of time. The size of the scatter markers are normalized by the sum of the peaks intensities to visualize the relative weight within the total PL response. Grey dashed line represents the laser excitation measured with the same streak camera. **d,** Energy-resolved fit of the PL emission. Shaded area represents the error bar area for the amplitude and lifetime fit, in brown and light blue respectively. We note a larger error of the fit from the emission with longer lifetime expected, as the time measurement range of the Streak camera becomes comparable with estimated lifetime, leading larger uncertainity[27].

PL in the time and energy domain. It is clear, that in the first few hundreds of picoseconds a high energy PL peak decays, leaving behind a lower energy long lifetime IX peak whose lifetime far exceeds the time range analysis of this measurement. We confirm this, by fitting the exponential decay for each energy, as shown in Fig. S12d. Here, we clearly see that high-energy emission features a lifetime of few hundreds of picoseconds, which increases to tens of nanoseconds in the lower energy case. Here, we specify that large uncertainty arises from the comparable measurement time-range and lifetime[27]. For better estimates of the long lifetime in the excitation region we point the reader to Fig. 3c and related discussion, where we measured the longer lifetime by TCSPC technique. Notably, the decay of the high energy peak happens in the same time domain where we observe fast exciton transport to remote recombination sites. This is a clear signature of the fast depopulation of high energy interlayer excitons from the excitation region, which have been also detected in this work *via* galvo-scanning imaging in the same time domain in remote regions of the sample. We note that, the energy difference found between these two species of exciton, does not match the singlet-triplet interlayer exciton

energy, which amounts to 25meV[28–30], and would not be expected in R-type stacked heterostructures, such as the one understudy. The energy difference we find here, reaches 45meV at peak excitation times, which is also much smaller than the estimated exciton barrier height of moiré potential in R-type $WSe_2/MoSe_2$, i.e. 90meV[16,31–33], and smaller than the expected energy difference between $K_{CB} - K_{VB}$ and $\Lambda_{\mathrm{CB}} - K_{VB}$ transitions, which would amount to 55 meV[34]. Therefore, we expect this energy difference to arise from many-body effects and from the specific reconstructed lattice potential at the excitation region.

We now perform electro-modulation spectroscopy on the sample to study the absorption energy of interlayer exciton within our structure, as already reported by Barré et al[34]. We have shown in Fig. 2d of the main text a subset of the heterodyne signal arising from interlayer exciton absorption. Here we show a larger set of data across the gated part of the sample to study the change in interlayer exciton absorption across the heterostructure (Fig. S13a). We observe large variations of the interlayer exciton absorption energy across the heterostructure, featuring a distribution of IX absorption energies that spans 40 meV (Fig. S13b). Notably, the lattice-induced potential gradients in our sample are comparable in magnitude to the intrinsic energy difference between high- and low-energy excitons. This suggests that excitonic drift driven by these gradients provides a plausible explanation for the rapid transport of excitons from the excitation region to remote recombination sites.

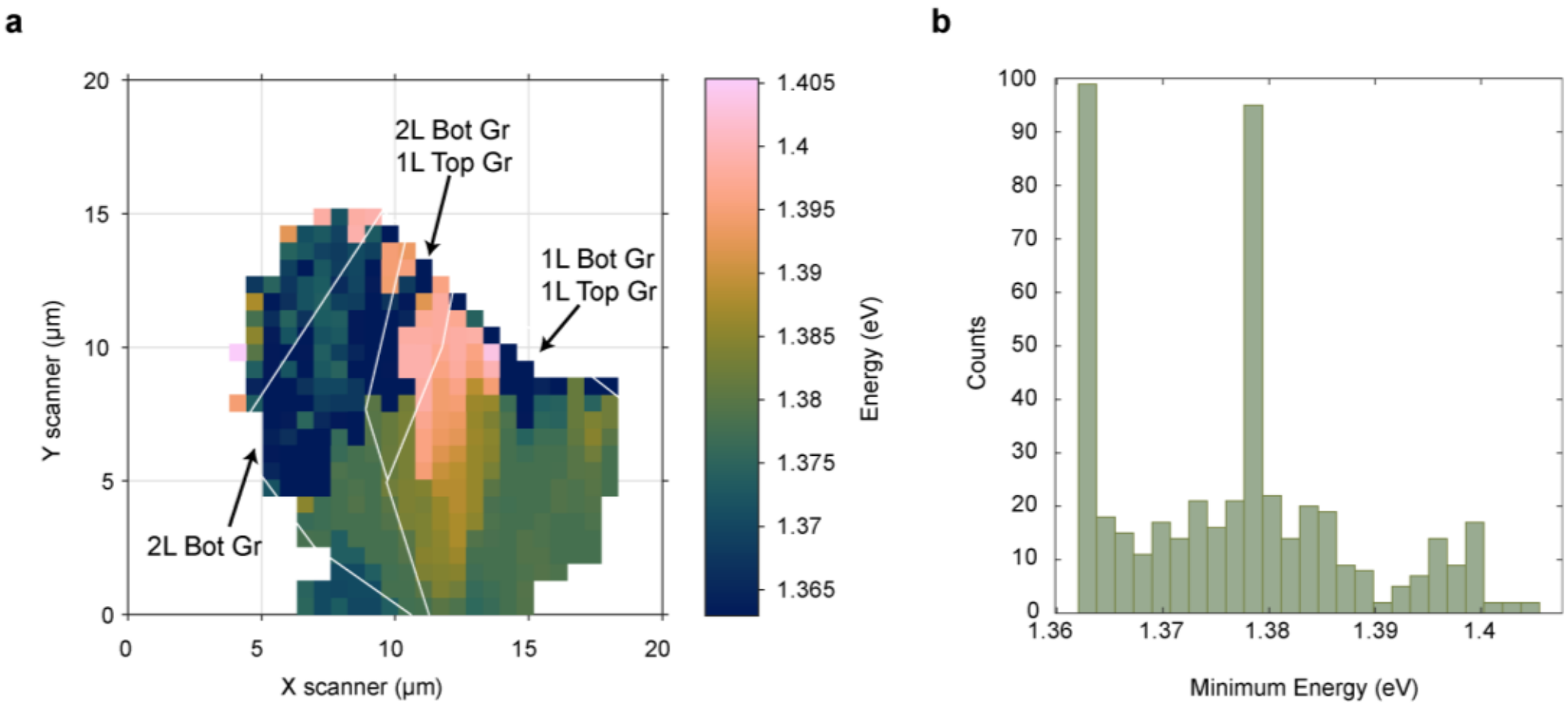

Figure S13 – Electromodulation spectroscopy map. **a**, Spatial map of the energy absorption dip. The white arrow corresponds to the arrow in Fig. 2b for the linescan of Fig. 2a. **b,** Histogram of the energy absorption dip across the gated heterostructure, showing a large range of energy absorption spanning roughly 40 meV.

9. FEM Simulation of IX Propagation

In order to further understand the dynamics of IX propagation in an inhomogeneous excitonic potential, we perform 1D axially-symmetric FEM simulation solving the PDE which describes exciton motion. The equation has the following formulation:

$$\frac{\delta n}{\delta t} = D_0 \nabla^2 n - \nabla \cdot \big(\mu n \nabla (U_{ext} + U_0 n)\big) - \frac{n}{\tau} - R_A n^2 + S(r,t) \tag{7}$$

Where, n is the exciton density, t is the elapsed time, D_0 is the diffusivity coefficient, μ is the field-effect mobility, U_{ext} is the potential energy landscape estimated in previous section, U_0

is the interaction energy, τ is the exciton lifetime, R_A is the Auger recombination coefficient and S is the gaussian shaped excited exciton density estimated in previous section. The Auger recombination and the interaction energy terms have been taken from ref [15], while we used the μ estimated from our fitting in Fig. 2c. In the FEM model, we have to input an actual electrostatic field, and we estimated this from the maximum spread of the excitonic potential energy, i.e. ~40meV, and the maximum distance at which we report remote recombination ie ~12 μm. This gives an average lower bound to the electrostatic field that is present in our sample, and will be affecting IX propagation. In Fig. S14 we present a comparison between our fit data and the computed normalized density at the same distances from the excitation region, studied in the experimental part of this work. We can clearly see that for small distances, the fast decay of IX density is very well matched in our model, strongly supporting the rapid escape of IX from the excitation region as an explanation of fast apparent interlayer exciton decay shortly after generation. As the distance is increased, a clear divergence between the rise of the density appears, we note that this might well be explained by the fact that we assume an average electrostatic field, while in the real experimental sample, this won't be the case, and larger electrostatic fields in shorter distances are likely to be present. A throughout characterization of this, is however outside the scope of this work. However, we note that at larger distances the time at which the modeled and fitted density and PL, respectively, converges again to similar time ranges, showing qualitatively good agreement. Furthermore, we note that the rapid escape of interlayer exciton from the excitation region, has important repercussion on confocal PL studies of IX. For example, estimation of the auger recombination coefficient from the power-dependent PL emitted from the excitation region will lead to an overestimation of non-radiatively recombined excitons, while in fact these are just moving outside of the detection area. Future studies shall keep this kind of dynamic into account for more accurate estimation of this kind of dynamic. Furthermore, in Fig. S15 we show the estimated IX density from the FEM 1/e area time behavior and the generated exciton density. It is clear, that exciton density stays above the Mott threshold density only for few hundreds of picoseconds after the laser pulse, further supporting the limited contribution of electron-hole plasma diffusion in this study.

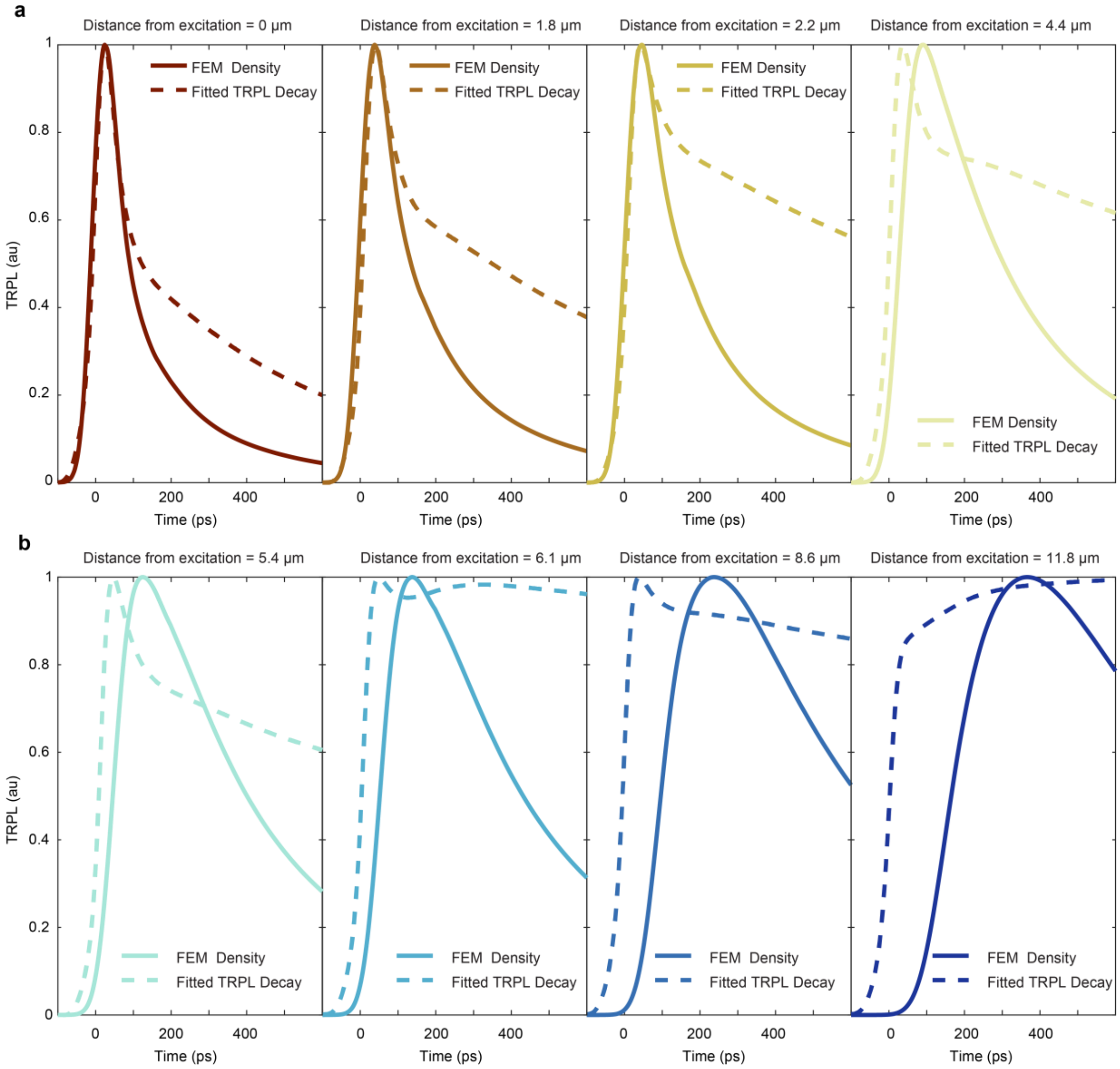

Figure S14 – Fit TRPL and FEM Model comparison. **a,b**, Spatial and time dependent density and TRPL signal simulated by FEM modeling and from experimental data respectively.

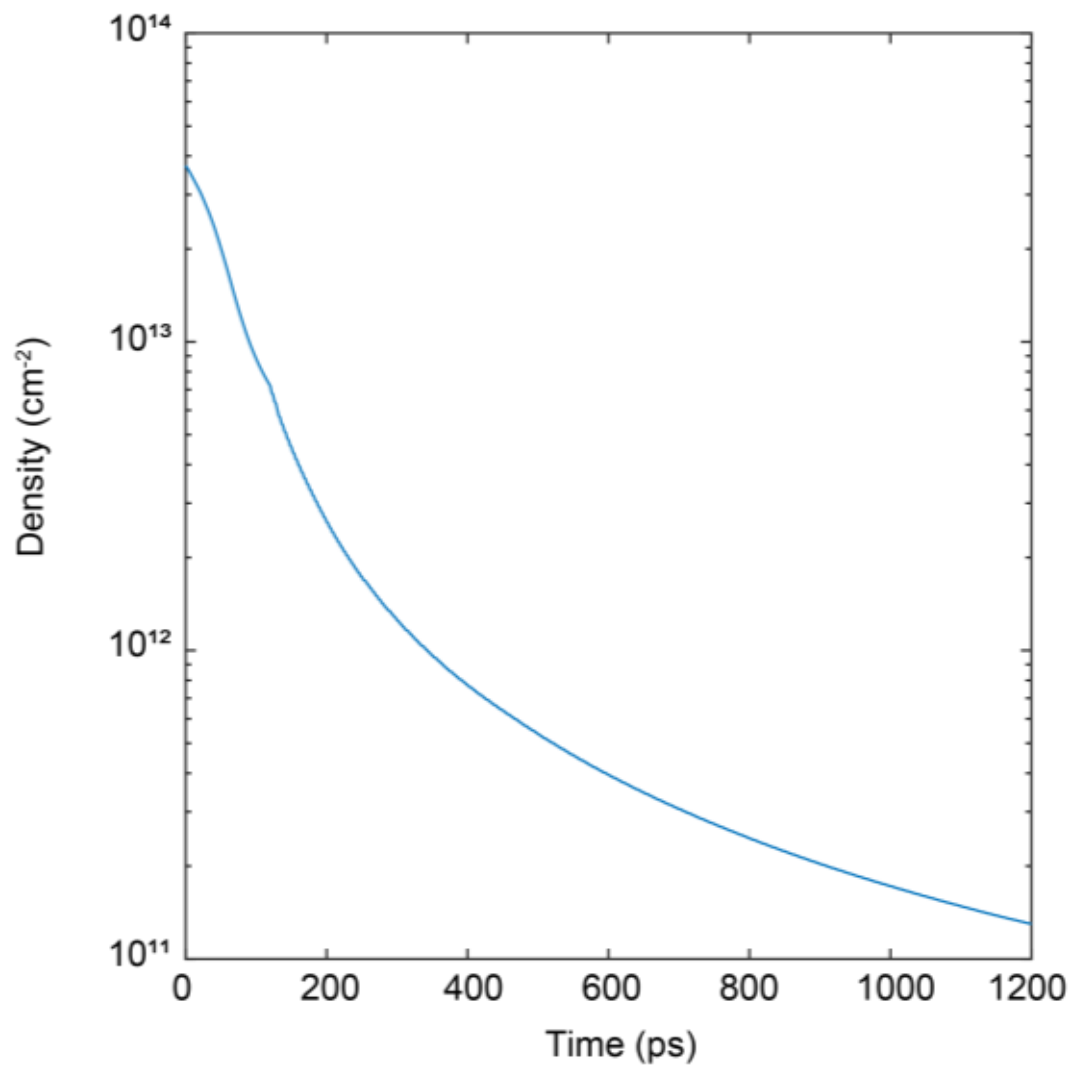

Figure S15 – FEM Estimated density. **a,** Estimated IX density as a function of time, estimated from the area expansion of IX and the generate exciton density.

10. Power-dependent IX transport

Here, we report on the excitation-dependent, time- and space-resolved photoluminescence study displayed in Fig. S16. In particular, Fig. S16a (S16b) shows the intensity-normalized (raw) time- and space-resolved PL traces acquired at the different optical excitation levels. Due to technical constraints, we are not able to collect enough signal in this configuration for measurements at lower excitation regimes. However, in this limited generated exciton regime, we clearly resolve a change in the collected signals as we increase the incident laser power. Specifically, we observe a delayed response of the TRPL signal at large distances from the excitation region. This is clear evidence of a decrease of the effective mobility as we increase the generated exciton density. As discussed in the main text, this can be attributed to the increase of the slower contribution to the overall interlayer exciton transport. In fact, any other known density-dependent or phonon-induced transport mechanism features slower transport diffusivities, than the one studied in this work. Therefore, larger generated exciton densities or larger optical excitation powers can increase their role in the total exciton transport mechanism, effectively decreasing the overall mobility and resulting in a delayed or slower TRPL response at larger distances from the excitation region.

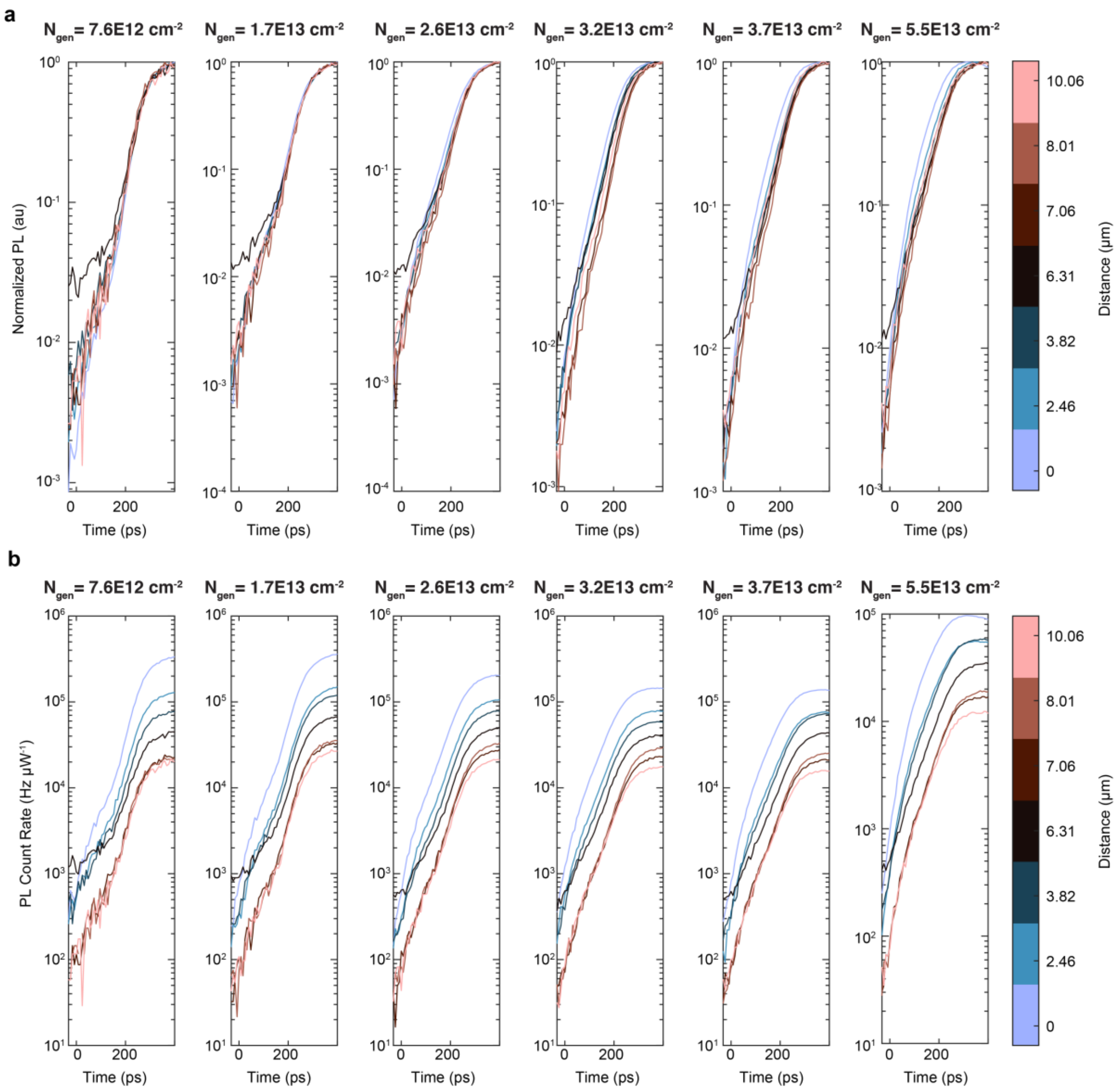

Figure S16 – Power-dependent time-resolved IX PL emission. a-b, Normalized (a) and non-normalized (b) TRPL data for increasing generation power densities. Different recombination spots are highlighted by their color coding, based on their distance from the excitation spot.

Via time-integrated PL imaging we estimate the average distance as a function of the cryostat temperature and the generated exciton density, shown in Fig. S17. This is an extension of the dataset shown in Fig. 4d, and doesn't feature any clear evidence of different excitonic phases that can be resolved *via* the presence of the long-range exciton transport. Together with power-dependent time-resolved data, this observation excludes superfluidity or hydrodynamic effects as origin of the fast interlayer exciton transport mechanism. In fact, in this case we would have observed a density-dependent increase of the transport speed, or clear phase regimes in Fig. S17.

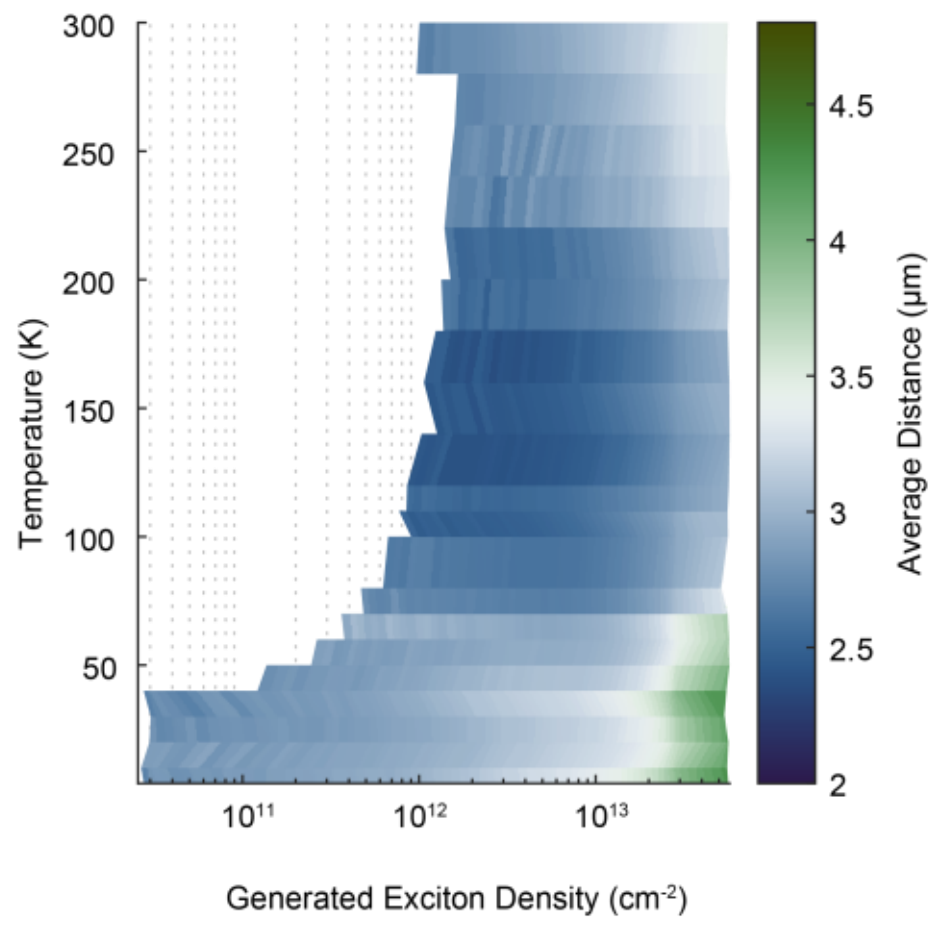

Figure S17 – Temperature- and power-dependent average distance. **a**, Average distance plotted against experimental temperature and generated exciton density estimated from time-integrated PL imaging. Blank region has been masked due to low PL counts and low SNR.

11. Experimental setup

In this work, we focus on the long-range diffusion of interlayer excitons, that feature highly inhomogeneous spatial recombination patterns. To achieve this, we exploit a galvo-scanning detection scheme in order to sweep the confocal detection across our detectors, and thus characterize interlayer exciton recombination far away from the excitation site. This technique allows us to reveal a new fast and long-distance interlayer exciton transport phenomena. The schematic representation of the setup employed in this study is shown in Figure S18. All the measurements are performed in a helium flow cryostat at a temperature of 4.6K unless otherwise mentioned. Pulsed laser excitation is performed with a supercontinuum white laser (NKT Photonics, FIU-25), whose spectral bandwidth is reduced through a variable bandwidth tunable filter (NKT Photonics, Varia). All the measurements have been performed with the variable filter set at 680nm central wavelength with 10nm bandwidth. CW measurements were performed instead using a solid-state diode laser, which can be driven both in pulsed and CW mode (Picoquant, LDH-IB-640-M), whose emission wavelength is 640nm. In the latter case, excitation is filtered with a short-pass filter with a threshold wavelength of 650nm. Throughout this work, we refer to steady-state or CW as the study conducted with CW excitation, while with time-integrated study we refer to imaging performed with pulsed excitation. In both configurations, the excitation is linearly polarized and focused on the sample with a 50x objective lens (Olympus LCPLN50XIR), focusing the laser light in a Gaussian spot with FWHM 1.3 μm. For micro-photoluminescence (μPL) spectral investigations, the emitted light is filtered by a 700 nm long-pass edge filter before being analyzed by a spectrometer (Princeton Instruments SpectraPro 500) and recorded using a CCD camera (Princeton Instruments, Blaze 400-HR/HRX). Photoluminescence spatial maps were acquired by scanning the objective position with a piezo scanning stage (Mad City Labs Nano-T). Spatial imaging of the interlayer exciton cloud has been performed by filtering the PL emission with an 850 nm long-pass edge filter and recorded with a CCD camera (Andor Ixon Ultra). The time-resolved analysis employed the supercontinuum white laser to generate pulses shorter than 50 ps at a 1MHz repetition rate. Emitted photons were detected by a single-photon counting module (MPD, PDM). The output of the APD is connected to a time-correlated single-photon counting module (PicoQuant, PicoHarp 300) with a 12 ps r.m.s. resolution recording the arrival time of each photon, with a set time bin of 4 (16) ps for rise time (lifetime) estimation. The primary temporal

constraint of this arrangement is the excitation pulse temporal FWHM, approximately 50 ps. Galvo scanning measurements have been performed directing the detection optical path to a galvo-scanner (Thorlabs, GVS012). Optical routing of the detection plane has been executed focusing the scanning signal with a scan lens (SL50-CLS2) on the tip of a multimode fiber with 50 µm core size. The other end of the fiber patch cable has been either connected to the same Single-Photon Counting Module (SPCM) for time and space resolved dataset or optically coupled to the spectrometer for galvo-scanning spectroscopy measurements. SHG measurements were performed using an 80MHz 180-fs Ti:Sa laser system (Coherent, Chameleon Ti:Sapphire) set at 900nm emission wavelength. The fs laser polarization has been set with a Glan-Thompson crystal polarizer (Thorlabs, GTH5M-B) and chromatically filtered with a long-pass 700nm edge filter.

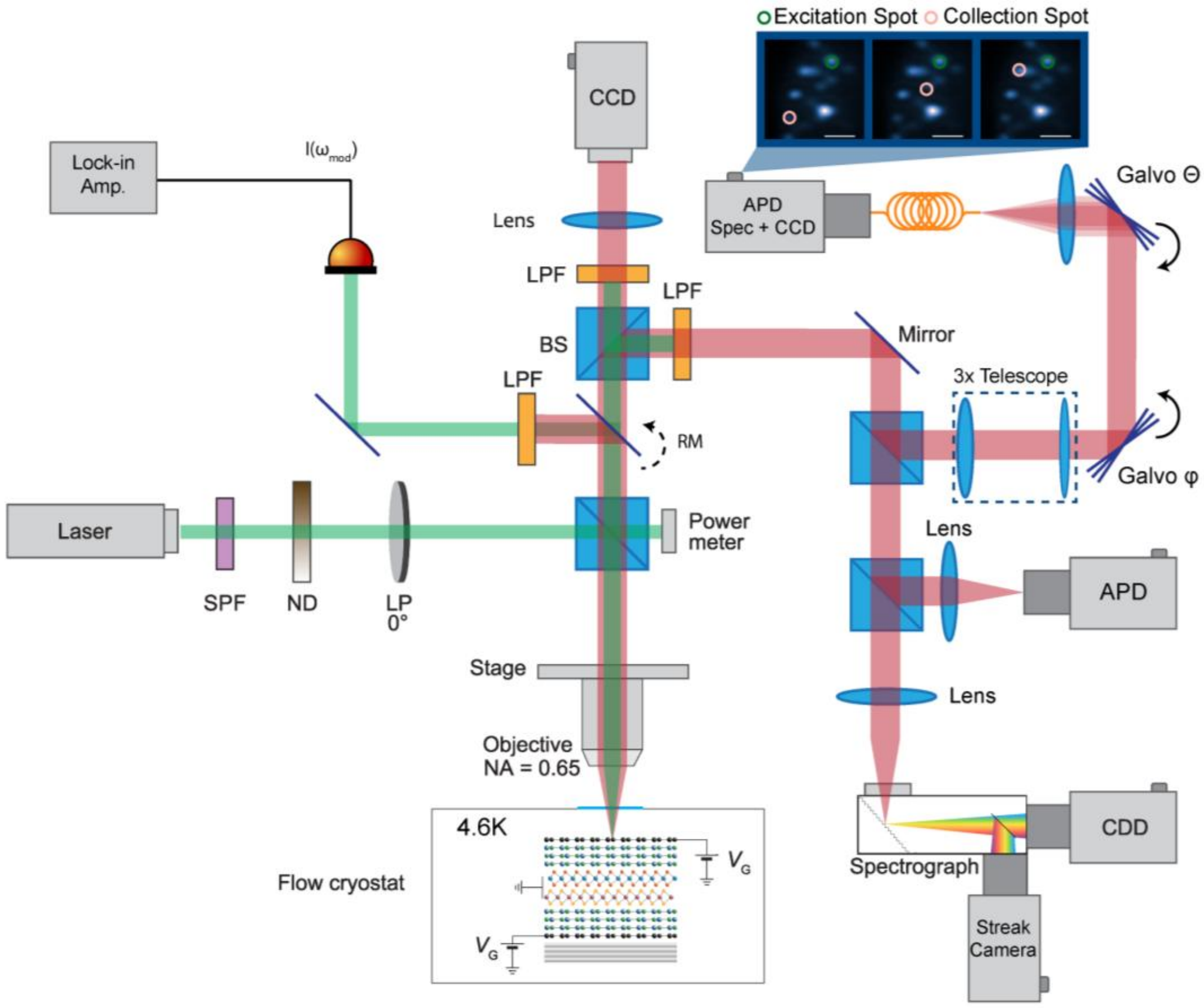

Figure S18 - Experimental setup schematics. Schematic view of the employed setup. Acronyms are respectively SPF for short-pass edge filter, ND for neutral density filter, LP for linear polarizer, BS for beamsplitter, LPF for long-pass edge filter, APD for avalanche photodiode, CCD for charge-coupled device and RM for removable mirror.

Linear Polarization for crystalline axis orientation was rotated with a lambda-half super-achromatic waveplate (SAHWP05M-700) mounted on a motorized rotation stage (Thorlabs, PRM1/MZ8). SHG spatial maps were performed splitting reflected excitation and SHG signal with a 650nm edge dichroic mirror. The reflected signal was recorded with a standard photodiode for spatial reflectance, while the SHG signal was analyzed with a linear polarizer, parallel to excitation polarization, and further filtered with a 500 nm edge short-pass filter before being recorded by our spectrometer system.

Electro-modulation spectroscopy measurements have been performed by applying a small sine voltage, typically 0.1V amplitude, to the top and bottom gate voltage. The sinusoidal voltage

at both gates has a phase offset of 90 degrees, therefore applying a net electric field. We use as lock-in amplifier and sine voltage generator a Stanford research SR860. The reflected laser intensity is measured by an amplified photodetector (Thorlabs, PDA36A2) and connected to the lock-in amplifier input. To perform wavelength-dependent electromodulation studies, we use our supercontinuum white laser coupled to a tunable acousto-optic filter (NKT Select), which provides chromatic linewidths of roughly 2.5 nm. Optical microscopy images and widefield PL images have been recorded in ambient with a commercial optical microscope (Olympus, BX51), while atomic force microscopy images have been acquired on a commercial Asylum Research Cypher S AFM Microscope.

References

(1) Hsu, W.-T.; Zhao, Z.-A.; Li, L.-J.; Chen, C.-H.; Chiu, M.-H.; Chang, P.-S.; Chou, Y.-C.; Chang, W.-H. Second Harmonic Generation from Artificially Stacked Transition Metal Dichalcogenide Twisted Bilayers. *ACS Nano* **2014**, *8* (3), 2951–2958. https://doi.org/10.1021/nn500228r.

(2) Jauregui, L. A.; Joe, A. Y.; Pistunova, K.; Wild, D. S.; High, A. A.; Zhou, Y.; Scuri, G.; De Greve, K.; Sushko, A.; Yu, C.-H.; Taniguchi, T.; Watanabe, K.; Needleman, D. J.; Lukin, M. D.; Park, H.; Kim, P. Electrical Control of Interlayer Exciton Dynamics in Atomically Thin Heterostructures. *Science* **2019**, *366* (6467), 870–875. https://doi.org/10.1126/science.aaw4194.

(3) Ciarrocchi, A.; Unuchek, D.; Avsar, A.; Watanabe, K.; Taniguchi, T.; Kis, A. Polarization Switching and Electrical Control of Interlayer Excitons in Two-Dimensional van Der Waals Heterostructures. *Nat. Photonics* **2019**, *13* (2), 131–136. https://doi.org/10.1038/s41566-018-0325-y.

(4) Ciarrocchi, A.; Tagarelli, F.; Avsar, A.; Kis, A. Excitonic Devices with van Der Waals Heterostructures: Valleytronics Meets Twistronics. *Nat. Rev. Mater.* **2022**. https://doi.org/10.1038/s41578-021-00408-7.

(5) Zhao, S.; Li, Z.; Huang, X.; Rupp, A.; Göser, J.; Vovk, I. A.; Kruchinin, S. Y.; Watanabe, K.; Taniguchi, T.; Bilgin, I.; Baimuratov, A. S.; Högele, A. Excitons in Mesoscopically Reconstructed Moiré Heterostructures. *Nat. Nanotechnol.* **2023**, *18* (6), 572–579. https://doi.org/10.1038/s41565-023-01356-9.

(6) Glazov, M. M.; Suris, R. A. Ultrafast Exciton Transport in van Der Waals Heterostructures. arXiv March 28, 2024. https://doi.org/10.48550/arXiv.2403.19571.

(7) LaGasse, S. W.; Proscia, N. V.; Cress, C. D.; Fonseca, J. J.; Cunningham, P. D.; Janzen, E.; Edgar, J. H.; Pennachio, D. J.; Culbertson, J.; Zalalutdinov, M.; Robinson, J. T. Hexagonal Boron Nitride Slab Waveguides for Enhanced Spectroscopy of Encapsulated 2D Materials. *Adv. Mater.* **2024**, *36* (7), 2309777. https://doi.org/10.1002/adma.202309777.

(8) Smith, D. A.; McKenzie, G.; Jones, A. C.; Smith, T. A. Analysis of Time-Correlated Single Photon Counting Data: A Comparative Evaluation of Deterministic and Probabilistic Approaches. *Methods Appl. Fluoresc.* **2017**, *5* (4), 042001. https://doi.org/10.1088/2050-6120/aa8055.

(9) Groma, G. I.; Heiner, Z.; Makai, A.; Sarlós, F. Estimation of Kinetic Parameters from Time-Resolved Fluorescence Data: A Compressed Sensing Approach. *RSC Adv.* **2012**, *2* (30), 11481–11490. https://doi.org/10.1039/C2RA21773B.

(10) Ginsberg, N. S.; Tisdale, W. A. Spatially Resolved Photogenerated Exciton and Charge Transport in Emerging Semiconductors. *Annu. Rev. Phys. Chem.* **2020**, *71* (1), 1–30. https://doi.org/10.1146/annurev-physchem-052516-050703.

(11) Chernikov, A.; Glazov, M. M. Exciton Diffusion in 2D van Der Waals Semiconductors. In *Semiconductors and Semimetals*; Elsevier, 2023; Vol. 112, pp 69–110. https://doi.org/10.1016/bs.semsem.2023.09.001.

(12) Devlin, J.; Husmeier, D.; Mackenzie, J. A. Optimal Estimation of Drift and Diffusion Coefficients in the Presence of Static Localization Error. *Phys. Rev. E* **2019**, *100* (2), 022134. https://doi.org/10.1103/PhysRevE.100.022134.

(13) Qian, H.; Sheetz, M. P.; Elson, E. L. Single Particle Tracking. Analysis of Diffusion and Flow in Two-Dimensional Systems. *Biophys. J.* **1991**, *60* (4), 910–921. https://doi.org/10.1016/S0006-3495(91)82125-7.

(14) Codling, E. A.; Plank, M. J.; Benhamou, S. Random Walk Models in Biology. *J. R. Soc. Interface* **2008**, *5* (25), 813–834. https://doi.org/10.1098/rsif.2008.0014.

(15) Wietek, E.; Florian, M.; Göser, J.; Taniguchi, T.; Watanabe, K.; Högele, A.; Glazov, M. M.; Steinhoff, A.; Chernikov, A. Nonlinear and Negative Effective Diffusivity of Interlayer Excitons in Moiré-Free Heterobilayers. *Phys. Rev. Lett.* **2024**, *132* (1), 016202. https://doi.org/10.1103/PhysRevLett.132.016202.

(16) Li, Z.; Lu, X.; Cordovilla Leon, D. F.; Lyu, Z.; Xie, H.; Hou, J.; Lu, Y.; Guo, X.; Kaczmarek, A.; Taniguchi, T.; Watanabe, K.; Zhao, L.; Yang, L.; Deotare, P. B. Interlayer Exciton Transport in $MoSe_2/WSe_2$ Heterostructures. *ACS Nano* **2021**, *15* (1), 1539–1547. https://doi.org/10.1021/acsnano.0c08981.

(17) Yuan, L.; Zheng, B.; Kunstmann, J.; Brumme, T.; Kuc, A. B.; Ma, C.; Deng, S.; Blach, D.; Pan, A.; Huang, L. Twist-Angle-Dependent Interlayer Exciton Diffusion in WS_2–WSe_2 Heterobilayers. *Nat. Mater.* **2020**, *19* (6), 617–623. https://doi.org/10.1038/s41563-020-0670-3.
(18) Sun, Z.; Ciarrocchi, A.; Tagarelli, F.; Gonzalez Marin, J. F.; Watanabe, K.; Taniguchi, T.; Kis, A. Excitonic Transport Driven by Repulsive Dipolar Interaction in a van Der Waals Heterostructure. *Nat. Photonics* **2022**, *16* (1), 79–85. https://doi.org/10.1038/s41566-021-00908-6.
(19) Wang, J.; Shi, Q.; Shih, E.-M.; Zhou, L.; Wu, W.; Bai, Y.; Rhodes, D.; Barmak, K.; Hone, J.; Dean, C. R.; Zhu, X.-Y. Diffusivity Reveals Three Distinct Phases of Interlayer Excitons in $MoSe_2$/WSe_2 Heterobilayers. *Phys. Rev. Lett.* **2021**, *126* (10), 106804. https://doi.org/10.1103/PhysRevLett.126.106804.
(20) Tagarelli, F.; Lopriore, E.; Erkensten, D.; Perea-Causín, R.; Brem, S.; Hagel, J.; Sun, Z.; Pasquale, G.; Watanabe, K.; Taniguchi, T.; Malic, E.; Kis, A. Electrical Control of Hybrid Exciton Transport in a van Der Waals Heterostructure. *Nat. Photonics* **2023**, *17* (7), 615–621. https://doi.org/10.1038/s41566-023-01198-w.
(21) Wang, J.; Ardelean, J.; Bai, Y.; Steinhoff, A.; Florian, M.; Jahnke, F.; Xu, X.; Kira, M.; Hone, J.; Zhu, X.-Y. Optical Generation of High Carrier Densities in 2D Semiconductor Heterobilayers. *Sci. Adv.* **2019**, *5* (9), eaax0145. https://doi.org/10.1126/sciadv.aax0145.
(22) Passler, N. C.; Paarmann, A. Generalized 4 × 4 Matrix Formalism for Light Propagation in Anisotropic Stratified Media: Study of Surface Phonon Polaritons in Polar Dielectric Heterostructures: Erratum. *JOSA B* **2019**, *36* (11), 3246–3248. https://doi.org/10.1364/JOSAB.36.003246.
(23) Passler, N. C.; Jeannin, M.; Paarmann, A. Layer-Resolved Absorption of Light in Arbitrarily Anisotropic Heterostructures. *Phys. Rev. B* **2020**, *101* (16), 165425. https://doi.org/10.1103/PhysRevB.101.165425.
(24) Zotev, P. G.; Wang, Y.; Andres-Penares, D.; Severs-Millard, T.; Randerson, S.; Hu, X.; Sortino, L.; Louca, C.; Brotons-Gisbert, M.; Huq, T.; Vezzoli, S.; Sapienza, R.; Krauss, T. F.; Gerardot, B. D.; Tartakovskii, A. I. Van Der Waals Materials for Applications in Nanophotonics. *Laser Photonics Rev.* **2023**, *17* (8), 2200957. https://doi.org/10.1002/lpor.202200957.
(25) Hsu, C.; Frisenda, R.; Schmidt, R.; Arora, A.; de Vasconcellos, S. M.; Bratschitsch, R.; van der Zant, H. S. J.; Castellanos-Gomez, A. Thickness-Dependent Refractive Index of 1L, 2L, and 3L MoS_2, $MoSe_2$, WS_2, and WSe_2. *Adv. Opt. Mater.* **2019**, *7* (13), 1900239. https://doi.org/10.1002/adom.201900239.
(26) Song, B.; Gu, H.; Zhu, S.; Jiang, H.; Chen, X.; Zhang, C.; Liu, S. Broadband Optical Properties of Graphene and HOPG Investigated by Spectroscopic Mueller Matrix Ellipsometry. *Appl. Surf. Sci.* **2018**, *439*, 1079–1087. https://doi.org/10.1016/j.apsusc.2018.01.051.
(27) Bouchet, D.; Krachmalnicoff, V.; Izeddin, I. Cramér-Rao Analysis of Lifetime Estimations in Time-Resolved Fluorescence Microscopy. *Opt. Express* **2019**, *27* (15), 21239–21252. https://doi.org/10.1364/OE.27.021239.
(28) Seyler, K. L.; Rivera, P.; Yu, H.; Wilson, N. P.; Ray, E. L.; Mandrus, D. G.; Yan, J.; Yao, W.; Xu, X. Signatures of Moiré-Trapped Valley Excitons in $MoSe_2$/WSe_2 Heterobilayers. *Nature* **2019**, *567*, 66–70. https://doi.org/10.1038/s41586-019-0957-1.
(29) Zhang, L.; Gogna, R.; Burg, G. W.; Horng, J.; Paik, E.; Chou, Y.-H.; Kim, K.; Tutuc, E.; Deng, H. Highly Valley-Polarized Singlet and Triplet Interlayer Excitons in van Der Waals Heterostructure. *Phys. Rev. B* **2019**, *100* (4), 041402. https://doi.org/10.1103/PhysRevB.100.041402.
(30) Brotons-Gisbert, M.; Baek, H.; Campbell, A.; Watanabe, K.; Taniguchi, T.; Gerardot, B. D. Moir\'e-Trapped Interlayer Trions in a Charge-Tunable WSe2/MoSe2 Heterobilayer. *Phys. Rev. X* **2021**, *11* (3), 031033. https://doi.org/10.1103/PhysRevX.11.031033.
(31) Blundo, E.; Tuzi, F.; Cianci, S.; Cuccu, M.; Olkowska-Pucko, K.; Kipczak, Ł.; Contestabile, G.; Miriametro, A.; Felici, M.; Pettinari, G.; Taniguchi, T.; Watanabe, K.; Babiński, A.; Molas, M. R.; Polimeni, A. Localisation-to-Delocalisation Transition of Moiré Excitons in WSe_2/$MoSe_2$ Heterostructures. *Nat. Commun.* **2024**, *15* (1), 1057. https://doi.org/10.1038/s41467-024-44739-9.
(32) Tran, K.; Moody, G.; Wu, F.; Lu, X.; Choi, J.; Kim, K.; Rai, A.; Sanchez, D. A.; Quan, J.; Singh, A.; Embley, J.; Zepeda, A.; Campbell, M.; Autry, T.; Taniguchi, T.; Watanabe, K.; Lu, N.; Banerjee, S. K.; Silverman, K. L.; Kim, S.; Tutuc, E.; Yang, L.; MacDonald, A. H.; Li, X. Evidence for Moiré Excitons in van Der Waals Heterostructures. *Nature* **2019**, *567* (7746), 71–75. https://doi.org/10.1038/s41586-019-0975-z.
(33) Yu, H.; Liu, G.-B.; Tang, J.; Xu, X.; Yao, W. Moiré Excitons: From Programmable Quantum Emitter Arrays to Spin-Orbit–Coupled Artificial Lattices. *Sci. Adv.* **2017**, *3* (11), e1701696. https://doi.org/10.1126/sciadv.1701696.
(34) Barré, E.; Karni, O.; Liu, E.; O'Beirne, A. L.; Chen, X.; Ribeiro, H. B.; Yu, L.; Kim, B.; Watanabe, K.; Taniguchi, T.; Barmak, K.; Lui, C. H.; Refaely-Abramson, S.; da Jornada, F. H.; Heinz, T. F. Optical Absorption of Interlayer Excitons in Transition-Metal Dichalcogenide Heterostructures. *Science* **2022**, *376* (6591), 406–410. https://doi.org/10.1126/science.abm8511.

(35) *Introduction to Solid State Physics, 8th Edition | Wiley*. Wiley.com. https://www.wiley.com/en-us/Introduction+to+Solid+State+Physics%2C+8th+Edition-p-9780471415268 (accessed 2025-07-12).
(36) Meneghini, G.; Brem, S.; Malic, E. Excitonic Thermalization Bottleneck in Twisted TMD Heterostructures. *Nano Lett.* **2024**, *24* (15), 4505–4511. https://doi.org/10.1021/acs.nanolett.4c00450.
(37) Richter, M. Theory of Interlayer Exciton Dynamics in Two-Dimensional Transition Metal Dichalcogenide Heterolayers under the Influence of Strain Reconstruction and Disorder. *Phys. Rev. B* **2024**, *109* (12), 125308. https://doi.org/10.1103/PhysRevB.109.125308.
(38) Psilodimitrakopoulos, S.; Mouchliadis, L.; Paradisanos, I.; Kourmoulakis, G.; Lemonis, A.; Kioseoglou, G.; Stratakis, E. Twist Angle Mapping in Layered WS2 by Polarization-Resolved Second Harmonic Generation. *Sci. Rep.* **2019**, *9* (1), 14285. https://doi.org/10.1038/s41598-019-50534-0.
(39) Psilodimitrakopoulos, S.; Mouchliadis, L.; Maragkakis, G. M.; Kourmoulakis, G.; Lemonis, A.; Kioseoglou, G.; Stratakis, E. Real-Time Spatially Resolved Determination of Twist Angle in Transition Metal Dichalcogenide Heterobilayers. *2D Mater.* **2020**, *8* (1), 015015. https://doi.org/10.1088/2053-1583/abbf88.
(40) Knorr, W.; Brem, S.; Meneghini, G.; Malic, E. Exciton Transport in a Moir\'e Potential: From Hopping to Dispersive Regime. *Phys. Rev. Mater.* **2022**, *6* (12), 124002. https://doi.org/10.1103/PhysRevMaterials.6.124002.
